# Distinct precursor landscape of subcutaneous and visceral fat in development and aging

**DOI:** 10.1016/j.celrep.2025.116706

**Published:** 2025-12-19

**Authors:** Frances Lin, Hei Sook Sul

**Affiliations:** 1 Department of Nutritional Sciences & Toxicology, University of California, Berkeley, Berkeley, CA 94720, USA

## Abstract

White adipose tissue (WAT) is the primary energy storage organ and can be categorized mainly into subcutaneous adipose tissue (SAT) and visceral adipose tissue (VAT). Although all WAT accumulates triglycerides to store excess energy, VAT is associated with pathological conditions, whereas SAT is considered beneficial for metabolic health. In fact, SAT and VAT are from distinct developmental origins. Moreover, within these depots, there is heterogeneity in developmental origin and in adipose precursor population. In various conditions, such as obesity and aging, adipose tissue undergoes distinct changes. In aging, pathogenic VAT tends to increase, whereas protective peripheral SAT is known to be reduced significantly, but the mechanism driving this depot-specific reduction in SAT is not well understood. Here, we review recent research on depot-specific adipose precursor heterogeneity in SAT and VAT and new and distinct adipose precursor populations arising in SAT and VAT during aging to bring differential changes in adipose mass.

## INTRODUCTION

White adipose tissue (WAT) is an essential organ in metabolic homeostasis, serving as the largest energy reservoir as well as secreting adipokines to affect various biological processes, including food intake. In contrast to WAT, brown adipose tissue (BAT) releases energy as heat via non-shivering thermogenesis to maintain body temperature. WAT is distributed in various depots in defined positions throughout the body, and the two largest depots can be divided broadly into subcutaneous adipose tissue (SAT) and visceral adipose tissue (VAT). SAT is located just beneath the skin, and VAT is located within the central body cavity surrounding internal organs, with some differences in humans and mice. In addition to SAT and VAT, WAT also exists as mammary adipose, dermal adipose, and bone marrow adipose tissue, developing independently with specialized functions.^1–4^

Under conditions of energy surplus, adipocytes in WAT synthesize and store triglycerides (lipogenesis). At times of increased energy demand or energy deprivation, these triglycerides are hydrolyzed into fatty acids (lipolysis) to be released into circulation. Additionally, dependent on the energy status, adipocytes secrete hormones with paracrine and systemic effects on various tissues, including the brain, pancreatic β cells, liver, skeletal muscle, and cardiovascular system, to regulate physiological functions, such as appetite, thermogenesis, and glucose and lipid metabolism.^5^ Thus, maintenance of optimal adipose tissue mass and function is essential for systemic health, its dysregulation contributing to the development of metabolic diseases, such as obesity, insulin resistance, and cardiovascular disease.^6–9^

Aging is often associated with impairment of WAT function and is coupled with the prevalence of metabolic diseases, such as obesity and type 2 diabetes. Importantly, while pathogenic VAT mass tends to increase, a depot-specific decrease in protective SAT is observed in aging, but the underlying mechanisms have not been well understood. Generally, VAT is associated with insulin resistance, and expansion of VAT is a major risk factor for type 2 diabetes and metabolic syndrome, whereas SAT is believed to be protective against metabolic diseases. Considering the differences in SAT and VAT, their effects on metabolic health, and their expansion during aging, it would be important to understand the developmental origins of adipose precursor populations and their heterogeneity and characteristics. This review will focus on the differences in VAT and SAT in their development through aging, based on studies mainly in mice and some in humans.

## ANATOMICAL DIFFERENCES BETWEEN SAT AND VAT

In mice, SAT is found mainly in the posterior inguinal (iWAT) region and the anterior axillary region. Humans develop SAT in the abdominal, gluteal, and femoral regions, and human abdominal SAT can be further divided into a superficial and a deep subcutaneous layer, a division that is absent in mice. Despite these differences, studies of mouse iWAT and human abdominal WAT produced largely comparable results. Importantly, the accumulation of VAT depots differs in mice and humans. VAT in mice is located in the perigonadal (often referred to as epididymal WAT in males and periovarian WAT in females), perirenal, pericardial, retroperitoneal, mesenteric, and omental regions. Humans lack perigonadal VAT and organize most of their VAT into the omental, mesenteric, epicardial, and perirenal depots. Omental WAT is the largest in humans, with mesenteric and perirenal WAT being sizable depots, whereas perigonadal WAT is the largest in mice, and omental and mesenteric WAT in mice are negligible in size. Studies in mice VAT may not accurately reflect the physiological function or response of human VAT, since anatomical location impacts immunomodulatory and metabolic functions.^10^ The distribution of SAT and VAT also vary by sexes. Females preferentially accumulate SAT depots, such as the gluteofemoral region, whereas males tend to accumulate VAT depots.

## CHARACTERISTICS OF SAT AND VAT

Differences in the metabolic function exist between healthy SAT and VAT. Adipocytes in VAT are more metabolically active and lipolytic, exhibiting higher levels of both basal and catecholamine-induced lipolysis to release fatty acids, but are less responsive to insulin’s antilipolytic effects.^11^ Thus, VAT was shown in mice to be preferentially mobilized during fasting and weight loss.^12^ Compared to SAT, a greater proportion of VAT mass was shown to decrease during human weight loss studies.^13^ The rapid mobilization of VAT may provide a quick source of energy for internal organs, which may be advantageous under certain conditions. For its active metabolism, VAT, compared to SAT, is more vascularized and immunologically dynamic, with a higher percentage of immune cells in VAT versus SAT.^14^ VAT thus plays an important role in the immune defense against pathogens, as well as inflammation, in mice and humans.^15,16^ Under homeostatic conditions, resident immune cells, such as macrophages and regulatory T cells (Tregs), produce anti-inflammatory cytokines that limit inflammation and help to maintain glucose homeostasis.^17^

SAT, compared to VAT, has “beige/brite” adipocytes with thermogenic capacity that can arise mainly during cold exposure, especially in mice, to use fatty acids and glucose for thermogenesis, promoting insulin sensitivity.^18^ In humans, BAT has been suggested to show either a classical brown or a beige signature, or both, depending on anatomical locations.^19–22^ More recent studies demonstrated human BAT to be similar to the BAT of mice that are subjected to conditions approaching human thermal and nutritional conditions^23^ and can be stimulated by β3-agonist, a dominant regulator of BAT activity.^24^

## SAT AND VAT IN OBESITY AND METABOLIC DISEASE

When energy intake chronically exceeds energy expenditure, WAT expands by increasing individual adipocyte size (hypertrophy), as well as through the formation of additional adipocytes recruited from the preadipocyte populations (hyperplasia). Remodeling of the extracellular matrix and angiogenesis are required for the healthy expansion of WAT and are regulated by acute hypoxic and inflammatory signals released in the tissue.^25,26^ During nutrient excess, WAT becomes dysfunctional through chronic inflammation and fibrosis, characterized by excessive accumulation of extracellular matrix proteins and tissue stiffening.^27,28^ Fibrosis in adipose tissue has been shown to impair adipogenesis and adipocyte function in mice,^29,30^ leading to insulin resistance, ectopic lipid accumulation, and metabolic disease.^31^ Similarly, transcriptome analysis of human WAT revealed that obesity induces the expression of genes of the fibrosis pathway.^27^

Compared to VAT, SAT releases higher levels of anti-inflammatory and appetite-suppressing adipokines, whereas VAT produces higher levels of pro-inflammatory cytokines to exert differential physiological effects.^32^ SAT has been shown to be protective against metabolic diseases. Transplantation of SAT, but not VAT, into visceral depots has been shown to improve insulin sensitivity, demonstrating the differences between VAT and SAT in mice.^33^ In this regard, the expansion of VAT in obesity stimulates the migration and differentiation of immune cells to a pro-inflammatory phenotype and reduces the differentiation into alternative macrophages.^23^ It has also been described that diminished adipogenic capacity and thus hypertrophy of VAT may be a dominant mechanism underlying insulin resistance in obesity.^33^ Hypertrophic adipocytes undergo higher rates of lipolysis, produce higher levels of inflammatory cytokines, and secrete lower levels of insulin-sensitizing adipokines, such as adiponectin, specifically in VAT in mice and humans.^34–36^ Differing immune activity between SAT and VAT may also support the pathogenic effects of VAT.^37^ Overall, while both SAT and VAT store energy as triglycerides, each possesses depot-specific functions and impacts health and disease.

## DEVELOPMENT OF WAT

Although different depots of WAT possess distinct metabolic features, all WAT depots contain lipid-storing adipocytes. Adipocytes, along with osteocytes, myocytes, and chondrocytes, can be differentiated from mesenchymal stem cells *in vitro*. The adipocyte population is determined by the recruitment of precursors of the stromal vascular fraction of adipose tissue for adipocyte differentiation, i.e., adipogenesis. Adipose precursors (including less committed adipose progenitors and more committed preadipocytes) arise from multipotent mesenchymal stem cells. *In vitro*, adipogenesis involves two steps: in response to adipogenic signals, the cell cycle is arrested, and precursors undergo adipogenic differentiation by a cascade of transcription factors. CCAAT/enhancer binding protein β (C/EBPβ) and C/EBPδ are induced early during differentiation and activate peroxisome proliferator-activated receptor γ (PPARγ) and C/EBPα.^38^ Bona fide adipose progenitors have been traditionally identified by certain markers, such as Pref-1 (also called Dlk1), PDGFRα, and CD24.^39–44^ Pref-1 inhibits adipocyte differentiation by upregulating Sox9 expression, which binds to the promoter regions of C/EBPβ and C/EBPδ genes, suppressing their transcription.^45^ Moreover, the Wnt signaling pathway inhibits adipocyte differentiation of mesenchymal stem cells through the suppression of C/EBPα and PPARγ.^46^

The total number of adipocytes in adipose tissue is set mainly during adolescence, and thus, changes in WAT mass mostly reflect alterations in adipocyte size due to changes in triglyceride content by hypertrophy.^47^ However, certain extrinsic factors, such as obesogenic signals and hyperphagia, can stimulate the proliferation and differentiation of adipose precursors.^48^ Additionally, the microenvironment of adipose tissue also affects adipogenesis and adipocyte function. During adipose expansion by hypertrophy or hyperplasia, adipocytes and stromal cells interact and adapt to support tissue plasticity.^49^ Extracellular matrix stiffness can regulate adipogenesis and lineage commitment in mesenchymal stem cells, and vascular remodeling is required for nutrient transport and adipocyte function, growth, and survival.^50–52^ Additionally, the immune system plays an important role in maintaining adipose homeostasis and function. Signaling from both innate and adaptive immune cells influences adipocyte size and function, lipid transport, and whole-body lipid homeostasis.^53,54^

Adipose precursors not only undergo adipogenesis to form new adipocytes but also affect other cell types in adipose tissue. Adipocyte precursors facilitate the formation of blood vessels through the secretion of pro-angiogenic factors, promoting endothelial cell proliferation and differentiation in mice and humans.^55^ In humans, CD34^+^ adipose stem cells expressed mesenchymal (CD90), pericyte (PDGFRβ), and smooth muscle (α-SMA) markers, contributing to the stabilization of the vasculature.^56^ Additionally, in response to inflammatory stimuli, preadipocytes in mice and humans have been observed to have higher expression of pro-inflammatory cytokines with decreased adipogenic capacity to exhibit macrophage-like phenotype.^57,58^ Pdfrα^+^ adipose precursors with high Cd9 expression were shown to contribute to inflammation and fibrosis of adipose tissue, particularly in obesity in mice, and were correlated with human visceral omental adipose fibrosis.^29^ A delicate balance of signaling between different cell types is required for healthy adipose tissue maintenance and function. Overall, these findings highlight that adipose precursors are more than just a regenerating pool for new adipocytes; they directly and indirectly influence adipogenesis and the adipose tissue environment, which may both be beneficial or detrimental when dysregulated during metabolic disease states.

## DEVELOPMENTAL ORIGIN OF SAT AND VAT

In mammals, WAT forms *in utero* in the peripartum period and throughout life, with various depots developing at specific and distinguishable pre- and postnatal times. In mice, preadipocytes in the inguinal fat pad are first to appear prior to birth and are not observed in other anatomical WAT depots until the postnatal period. During murine embryogenesis, Pref-1-marked cells first appear in the dorsal mesenteric region as early as embryonic day 10.5 (E10.5). These cells become lipid-laden adipocytes at E17.5 in the subcutaneous region, whereas visceral WAT develops after birth.^42^ Adipose tissue in humans develops first in the head and neck before the trunk and upper and lower limbs.^59^

Transcriptomic analysis of adipocytes as well as adipose precursors from the stromal vascular fraction (SVF) of SAT and VAT showed consistent differences in developmental gene expression in both rodents and humans.^60,61^ Preadipocytes and adipocytes of VAT express higher levels of HoxA5, HoxA4, HoxC8, Gpc4, and Nr2f1. Preadipocytes and adipocytes of SAT have higher levels of HoxA10, HoxC9, Twist1, Tbx15, Shox2, En1, and Sfpr2. These differences in gene expression appear to be intrinsic when cells were in culture and after differentiation *in vitro*, demonstrating that they are cell autonomous, independent of the microenvironment.

While differing characteristics of SAT and VAT have been recognized for many years, whether divergent developmental origin contributes to these differences is unclear. Regardless, several studies in mice support distinct developmental origins of the two WAT depots by lineage tracing. Progenitors/preadipocytes expressing Wilms Tumor 1 (Wt1) were identified in VAT but not in SAT or BAT.^62^ Wt1 was expressed in six different VAT depots in late gestation, and a subset of adipocytes of VAT arose from Wt1-expressing cells postnatally. This report also suggested that WAT depots associated with visceral organs were from a mesothelial layer that serves as a source of adipocyte precursors. However, Wescott et al. contradicted that Wt1 expression in adipose tissue was not limited to the mesothelium, and mesothelial cells were not a source of adipocytes in mice. Adipocyte progenitor markers, PDGFRα and Sca-1, were not expressed in Wt1^+^ cells in the mesothelium.^63^ By single-cell RNA sequencing (scRNA-seq), another study reported a population of Tcf21 lineage cells present exclusively in VAT, contributing to the development and expansion of VAT.^64^ However, in adult mice, Tcf21 inhibited adipogenesis via accentuation of Pref-1 expression. Interestingly, expression of Tcf21 was detected in PDGFRα^+^ mesenchymal progenitor cells in the mesothelium. Tcf21 was specific to PDGFRα^+^ mesenchymal progenitor cells, whereas Wt1 was also detected in PDGFRα^−^ cells. These conflicting reports may reflect potential biases from lineage tracing or functional differences of those genes during development versus homeostatic maintenance. Wt1^+^ mesothelial cells may be distinct from Wt1^+^ preadipocytes, and progenitor populations may also be heterogeneous with differing origins. In contrast, SAT has been reported to be derived from cells of a different embryonic lineage. Lineage tracing with Prx1-Cre, a homeobox transcription factor expressed in embryonic limb and bud mesenchyme, labeled adipocytes in posterior SAT.^65,66^ A similar study using mT/mG mice crossed with Myf5-Cre known to be active in muscle and interscapular BAT, also labeled cells of anterior subcutaneous and renal WAT, but not iSAT or perigonadal VAT.^67^

Interestingly, the ability of cell types other than adipose precursors has been reported to differentiate into adipocytes. Endothelial cells from capillary sprouts of adipose tissue expressed Zfp423, known as a transcription regulator of preadipocyte determination.^68^ Lineage tracing with endothelial-specific promoter VE-cadherin showed that cells of endothelial origin, in response to PPARγ activation, lose endothelial characteristics and form structurally and biochemically defined white adipocytes.^69^ In addition, neuroepithelial cells from mouse embryonic stem cells were reported to be capable of adipogenic differentiation *in vitro*, and neural crest marker Sox10 promoter-driven YFP could label adipocytes in a subset of facial adipocytes between the salivary gland and ear.^70,71^ Cells expressing smooth muscle lineage markers, such as Acta2 (α-SMA) and Myh11, were also reported to differentiate into beige adipocytes.^72,73^ Overall, the studies on the origins of SAT and VAT precursors indicate their heterogeneity, yet they are not fully understood. Additionally, these studies may indicate heterogeneous developmental origins even in a single adipose depot. Whether these developmental origins result in functional differences among adipocytes within a depot requires further investigation.

## HETEROGENEITY OF ADIPOSE PRECURSORS OF SAT AND VAT

Given the heterogeneity in the developmental origins of SAT and VAT adipocytes, bona fide adipose precursors of each depot may have distinct characteristics. Indeed, intrinsic differences in the adipose precursor populations in SAT and VAT were reported.^74–76^ Compared to VAT precursors, adipose precursors in SAT were reported to be more capable of adipogenic differentiation, resulting in hyperplasia, whereas VAT precursors favor enlargement of cell size via hypertrophy.^47,76,77^ Wolfrum and colleagues showed that VAT had lower differentiation capacity independent of adipocyte precursor number in mice.^78^ By mass spectrometric analysis of secreted factors from SVF of SAT and VAT, these researchers identified decorin (Dcn) and Sparc-like 1 (Sparcl1) to be secreted by visceral preadipocytes to inhibit adipocyte differentiation. However, these results have been challenged, as reports have shown higher hyperplasia of VAT compared to SAT in mice.^48,79^ Differences may be due to anatomical WAT organization and heterogeneous stromal populations. Additionally, different growth factors have been shown to regulate adipocyte precursor differentiation and adipocyte numbers in a depot-specific manner.^80^

Recent scRNA-seq and single-nucleus RNA sequencing analyses have attempted to elucidate heterogeneity of adipose precursor populations in each depot in mice and humans^81–89^ (Table 1). A DPP4^+^ precursor population was shown in both SAT and VAT to express stem-related genes, such as Ly6a (Sca-1), Dpp4, Cd34, and was predicted to provide a source for committed preadipocytes.^81–83,88,89^ This population was highly proliferative *in vitro* and refractory to adipogenesis, lacking PPARγ expression with low expression of adipocyte markers. These cells were multipotent and had the ability to differentiate into adipocytes or osteocytes when treated with adipogenic or osteogenic cocktail, respectively. Anti-adipogenic transforming growth factor β (TGF-β) and Wnt signaling pathways were enriched in these cells, and treatment with TGF-β increased the expression of genes, such as Wnt10b and Cox2, and suppressed the expression of adipogenic markers. Conversely, TGF-β receptor inhibition of DPP4^+^ progenitor cells induced adipogenic markers. While the DPP^+^ cell population was detected in both SAT and VAT, VAT had a lower proportion of DPP4^+^ cells compared to other preadipocyte populations, potentially implicating its involvement in the reduced regenerative capacity of VAT. A DPP4^+^ cell population was also detected in human adipose tissue with similar higher proliferative activity and lower adipogenic potential.^82^

A second population, considered to be of a more committed preadipocyte population, was found in both SAT and VAT in mice and humans. This population was marked by the expression of Icam1 and Aoc3, as well as several collagens and extracellular matrix remodeling factors.^81–84,88^ These preadipocytes were shown to express classic adipocyte markers, such as Pparγ, Lpl, and Cd36, with a high degree of adipogenic potential *in vitro* and upon transplantation *in vivo*. Unlike adipose stem cells, preadipocytes had lower proliferation with higher adipogenic capacity and could differentiate into adipocytes even when treated with insulin only, in place of the complete adipogenic cocktail. Bioimaging studies indicated this population to be located proximally to the adipose vasculature. Human preadipocytes resembling this mouse preadipocyte population were identified by the expression of ICAM1, PPARγ, and GGT5, and by their low proliferation and high adipogenic capacity.^82^ A second group further separated ICAM1 precursors into ICAM1^+^CD44^high^ and ICAM1^+^CD44^low^ adipose precursor populations based on commitment to adipocyte differentiation, with ICAM1^+^CD44^high^ precursors at a less committed stage.^90^

A population named adipogenesis regulatory cells (Aregs), characterized by the expression of Cd142 and Abcg1, was reported in both SAT and VAT in mice and humans.^81,82,88^ Aregs were not only refractory to adipogenesis *in vitro* but also inhibited differentiation of preadipocytes via Spink2 secretion. Human CD142^+^ Aregs were also detected and shown to be refractory to adipogenic differentiation, judged by lipid accumulation and marker gene expression, and displayed anti-adipogenic paracrine signaling. A significantly higher proportion of CD142^+^ABCG1^+^ cells was found in visceral SVF, compared to the subcutaneous SVF, and its proportion increased in both depots in obese versus lean adipose. However, there has been controversy in the Areg population, as other studies found CD142^+^ cells to be fully adipogenic and not to affect cell differentiation of adipose precursors.^82,84^ The discrepancy may be due to differences in cell sorting strategies, highlighting the possible biases that may arise from cell population selection before characterization.

A unique VAT-specific population in mice marked by Ly6c^+^ PDGFRβ^+^ was identified and named fibro-inflammatory progenitors (FIPs) due to its fibrogenic and inflammatory phenotype.^86^ This population was isolated from PDGFRβ^+^ perivascular cells, and the population increases upon high-fat feeding. FIPs expressed adipose stem cells markers, such as Dpp4, as well as those related to TGF-β signaling pathway, and were more resistant to adipogenesis than Ly6c^−^PDGFRβ^+^ precursor subpopulations of DPP4^+^ progenitor cells. However, this population diverged from the previously mentioned DPP4^+^ progenitor cells in its pro-inflammatory and anti-adipogenic effects. FIPs expressed cytokines, such as IL-6, CXCL2, and CXCL10, and could activate macrophages *in vitro*. These cells were reported to be regulated by the NR4A nuclear receptor family, which has been implicated previously in inflammation and adipogenesis.^93^ While FIPs were shown to have anti-adipogenic properties, the exact factors involved in this process have yet to be identified. Whether a human FIP population exists is currently unknown.

PDGFRα, Sca1, and CD81 expressing beige adipose precursors were identified in the subcutaneous iWAT of mice, giving rise to beige adipocytes expressing high levels of Ucp1 and Cited1. Ablation of Cd81 in the SAT of mice caused diet-induced obesity, insulin resistance, and adipose tissue inflammation.^92^ While previous studies suggested VAT to be resistant to the formation of beige adipocytes,^94,95^ beige adipose precursor population in VAT has also been reported. Thus, an adipose progenitor population that differentiates into beige adipocytes upon β3-adrenergic receptor activation was identified in the epididymal VAT of mice.^83^ Additionally, a VAT-specific adipose progenitor population originated from the mesothelium, named VPM, as VAT progenitors from mesothelium origin were also identified in mice.^87^ VPMs were Cd34^+^ and Wt1^+^ and expressed mesothelial markers Upk3b and Wt1. They had high mitochondrial gene expression and were postulated to differentiate into beige adipocytes. VAT in humans is also reported to be able to undergo beiging and the induction of thermogenic genes in the perivascular adipose tissue to attenuate inflammation and pathological vascular inflammation in acute aortic dissection.^96^ In addition, in hypermetabolic/catabolic conditions, such as in cancer cachexia and burn patients or in severe adrenergic stress, beiging of SAT was reported in humans.^97–99^

**Figure 1.**
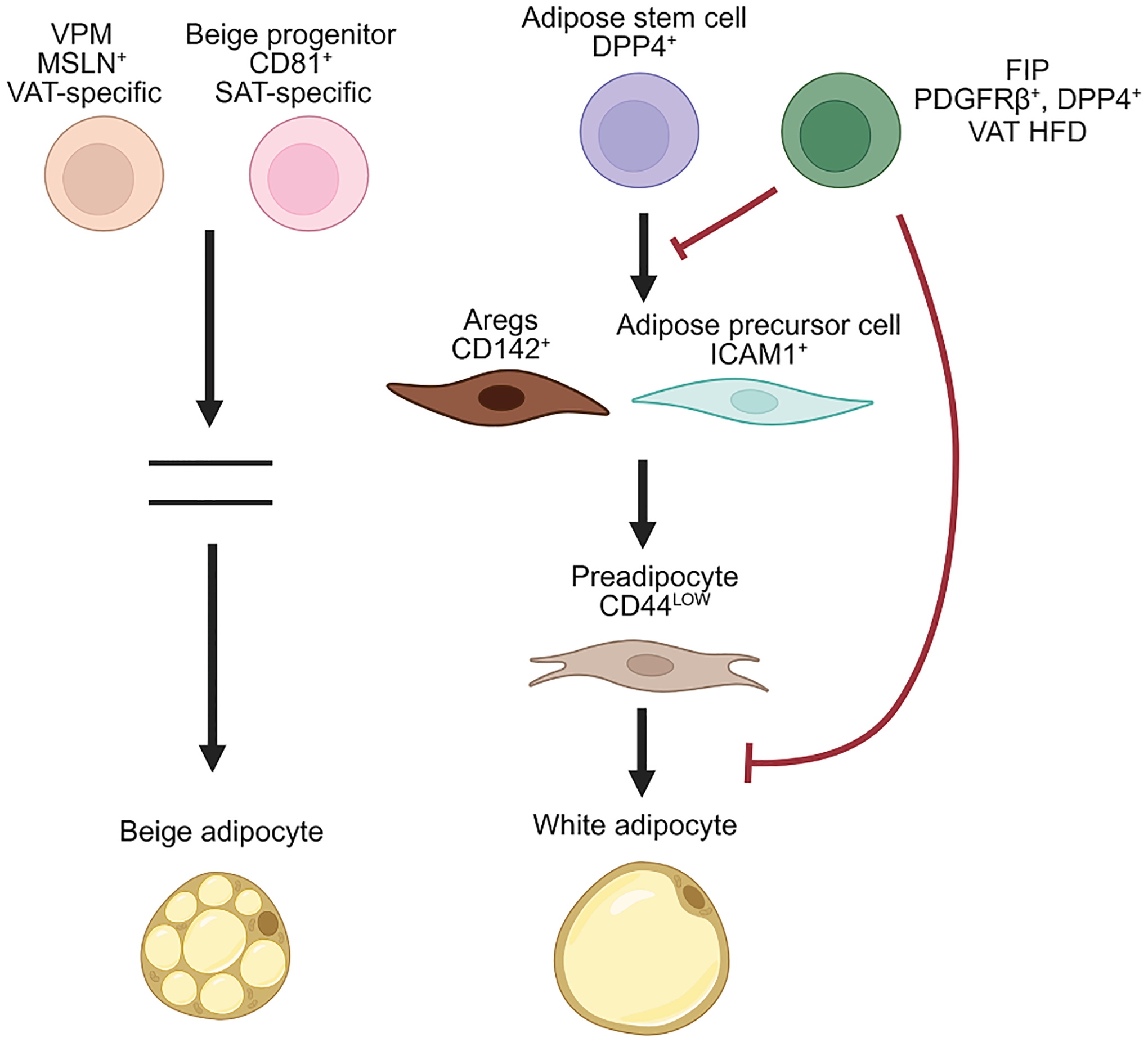
Adipose precursor heterogeneity in SAT and VAT There is heterogeneity in adipose precursor populations in subcutaneous adipose tissue (SAT) and visceral adipose tissue (VAT), with a hierarchy of adipose precursor populations during white adipocyte differentiation. A DPP4^+^ population of adipose stem cells in SAT and VAT is a less committed multipotent progenitor that is highly proliferative and more resistant to adipogenesis. ICAM^+^ and Aregs are more committed adipose precursor populations in SAT and VAT with enrichment of genes involved in adipocyte differentiation. The adipogenic potential and paracrine anti-adipogenic effects of Aregs are still under debate, with conflicting reports. A further committed preadipocyte population is found in SAT and VAT that expresses low levels of CD44, as compared to less committed CD44^high^ populations. PDGFRβ^+^ and DPP4^+^ FIPs have been shown to emerge in VAT during high-fat diet feeding; they are refractory to adipogenesis and exert paracrine anti-adipogenic effects on neighboring precursor cells. Beige adipocyte precursor heterogeneity is also observed. A CD81^+^ beige adipocyte progenitor is detected only in SAT, and MSLN^+^ VPMs are identified to be VAT-specific. However, the commitment to differentiation and its markers are still unknown.

Adipocyte populations arising from different adipose precursors in each depot may result in SAT and VAT having distinct functions (Figure 1). The existence of CD81^+^ beige progenitors in SAT may also explain how SAT, in comparison to VAT, favorably undergoes beiging during cold exposure in humans and mice.^94,100,101^ Additionally, FIPs found in VAT may explain the lower adipogenic potential and increased fibrosis of VAT in metabolic disease. However, the heterogeneity of adipose precursors and their distinct functions are still not fully understood. The identification of depot-specific progenitor populations in SAT and VAT would require lineage tracing and better identification of specific markers. Moreover, it is unclear what factors preferentially influence the development of certain precursor populations and how metabolic stress or aging may alter these populations. In addition, the interaction of adipose precursor populations with other stromal cell populations may influence the metabolic and pathological differences observed in SAT and VAT. Finally, as with adipose precursors, adipocyte populations have been shown to be heterogeneous even within a single depot. Depot-specific adipocyte subpopulations with differing functions^102,103^ have been identified, and these subpopulations may play a role in the differences observed between SAT and VAT. For example, Wolfrum et al. revealed a subpopulation of adipocytes that could regulate thermogenesis in SAT of both mice and humans.^104^ Spatially resolved transcriptional profiling along with scRNA-seq identified 3 distinct human adipocyte subpopulations in SAT with distinct sensitivity to insulin, termed Adipo^LEP^, Adipo^PLIN^, and Adipo^SAA^.^102^ In addition, a human VAT-specific adipocyte population with a relatively high expression of mitochondrial and ribosomal genes was also reported.^103^ It is plausible that distinct adipocyte populations may be differentiated from different adipose precursors.

## ADIPOSE TISSUE IN AGING

A recent transcriptional analysis showed that WAT is one of the first tissues to undergo age-dependent remodeling and may be a critical driving force for organismal aging.^105,106^ In aging, adipose tissue undergoes changes in fat depot redistribution, adipose precursor populations, accumulation of senescent cells, and dysregulation of immune cells. Redistribution of WAT is characterized primarily by accumulation in the visceral region, VAT, along with ectopic deposition of lipids in other organs. Thus, the mass of pathogenic VAT tends to increase with adipocyte hypertrophy, chronic low-grade inflammation, and fibrosis in humans,^107^ whereas the mass of protective SAT is reduced significantly in both mice and humans.^108^ Studies have also indicated aging-associated fibrosis in both SAT and VAT.^109^ In addition, fibrosis in SAT in humans was reported to be associated positively with visceral adiposity.^110^

On a cellular level, the main characteristics of adipose tissue aging include changes in the adipogenic potential of precursors, accumulation of senescent cells, and increased adipose tissue inflammation. In general, the number of adipose progenitors and their proliferative and adipogenic capacity is accepted to decline with increasing age.^79,111–113^ Alt et al. reported a 2-fold decrease in the proliferation rate of adipose precursors of both abdominal and subcutaneous adipose in aged compared to young patients.^114^ A decrease in the expression of adipogenic transcription factors, such as PPARγ, C/EBPδ, and C/EBPα, and an increase in anti-adipogenic transcription factors, such as C/EBPβ-LIP and CHOP, has been observed.^115^ Additionally, adipose aging has been associated with cellular senescence in general and the loss of the ability of preadipocytes to differentiate into adipocytes in both mice and humans.^116^ Cellular senescence is characterized by cell cycle arrest and secretes an array of cytokines, chemokines, proteases, and growth factors, known as senescence-associated secretory phenotype (SASP).^117^ Senescent human and mouse preadipocytes have impaired adipogenic potential and accumulate in adipose tissue with aging.^118,119^ These cells impact the neighboring adipose precursors by secreting inflammatory factors and impairing adipogenesis.^120^ Changes in immune populations, such as lymphocytes, have also been observed.^121^ Adipose tissue in old mice expresess higher levels of inflammatory cytokines, such as IL-1, IL-6, tumor necrosis factor alpha (TNF-α), as well as lipid inflammatory mediator COX2.^122^ Preadipocytes have also been shown to release inflammatory cytokines, such as TNF-α, to impair adipogenesis^123^ and disrupt insulin signaling^124^ in mice. Thus, chronic inflammation accompanies adipocyte hypertrophy and insulin resistance, resulting in WAT dysfunction and metabolic disease in aging.

## DIFFERENCES IN ADIPOSE PRECURSORS OF SAT AND VAT DURING AGING

Importantly, changes in VAT and SAT differ during aging in that VAT tends to increase, whereas SAT mass is reduced significantly in both mice and humans.^111,125^ While chronic positive energy intake may contribute to the increase in VAT mass, how SAT mass decreases during aging has not been well understood. Given the heterogeneity of adipose precursors in each depot, changes in certain populations may bring changes in SAT and VAT in a depot-specific manner.

## SENESCENCE OF ADIPOSE PRECURSORS IN SAT AND VAT DURING AGING

Senescence has been observed in both SAT and VAT, but there appears to be depot-specific differences in senescent cell accumulation during aging. In humans, SAT during aging was reported to be associated with increased adipose progenitor cell senescence and reduced adipogenesis.^126^ Telomere length, a marker of cellular senescence, was also reported to be shorter in total human SVF^83^ and mice adipose progenitor cells^84^ of SAT than VAT, suggesting SAT is more prone to age-related injuries. Moreover, human VAT senescent cells in total adipose tissue were reported to express more inflammatory cytokines than SAT senescent cells.^127^ In addition, van Deursen et al. reported tissue-specific induction of p16 in adipose tissue and skeletal muscle in an accelerated aging in BubR1 hypomorphic mice. The p16 induction total SAT was attributed to cellular senescence, and these mice exhibited an early loss of SAT and a lower proportion of bromodeoxyuridine-positive cells in adipose tissue compared to their wild-type cohort.^128^ BubR1 hypomorphic mice developed a SASP phenotype in iSAT as they age, and targeted elimination of p16-positive cells rescued this phenotype.^129^ However, p16 induction in aging was also shown in VAT, primarily in Cd31^+^ endothelial cells, and to a lesser extent in adipocytes and macrophages in mice.^130^ In addition, a recent study suggested that p27 and cdk2 could contribute to depot-specific differences in aging, as a reduction of p27 and cdk2 was detected in SAT, but not in VAT, during aging in mice. However, other senescence markers, such as p21 and p57, did not change significantly in SAT but increased in VAT of aged mice.^131^ Cyclin-dependent kinases and kinase inhibitors, including p21 and p27, are closely related to cellular senescence and have also been implicated in hyperplasia of adipose tissue.^132^

A study in humans investigating SAT in Werner syndrome, a hereditary premature aging disorder characterized by VAT accumulation and SAT lipoatrophy, showed early replicative senescence and a significant increase in the expression of SASP markers in adipose stem cells of SAT.^133^ Moreover, human senescent adipose stromal cells had noticeably decreased adipogenic potential and could inhibit adipogenesis of the surrounding non-senescent progenitors.^134^ Activin A expressed from adipose progenitors in older mice compared to young mice was higher in both circulation and adipose tissue, and the secretion of activin A by senescent cells in humans could impair adipocyte differentiation.^135^ Suppression of activin A secretion by inhibition of JAK/STAT pathway rescued adipogenic potential.^120^ However, other components of SASP, such as IL-6, TNF-α, and interferon gamma, may also inhibit the differentiation of neighboring precursor cells.^136^

Studies have shown the modulation of sirtuin expression in adipose tissue during aging, which may lead to early cell senescence. Sirtuins have been implicated as essential regulators of obesity-associated adipose remodeling, and recent reports showed that the expression and activity of sirtuins decrease in adipose tissue of mice during aging,^137,138^ but depot differences in sirtuin expression during aging in adipose tissue have not been well studied. Sirt7 mRNA and protein levels in rats decreased during aging in total SAT depots but not VAT depots.^139^ Sirt7 has previously been shown to promote adipogenesis in mice by inhibiting Sirt1 and rescuing Sirt1 repression of PPARγ.^140^ However, Sirt1 has been shown to either attenuate or enhance adipogenesis under specific conditions.^141,142^ In addition, decreased Sirt1 expression was also observed in adipose stem cells of SAT in aging female human patients, leading to suppression of adipocyte differentiation and decreased adiponectin levels.^143^ It is not known what drives these expression changes, considering the many functions of sirtuins and their ubiquitous expression. Further investigation may lead to a better understanding of the role of sirtuins in changes in adipose tissue mass during aging.

Overall, both intrinsic and microenvironmental differences between depots may influence depot-specific alterations in adipose mass during aging. However, the differences in senescence between adipose precursor cells of SAT and VAT and how they affect adipose tissue and function remain unclear. It is not certain whether these differences observed in adipose precursor cells contribute to depot-specific differences or rather are a product of their environment, as most reported studies were on total adipose tissue. Moreover, it is unclear if all adipose precursor populations exhibit similar levels of senescence during aging. Further studies are needed to identify senescence markers specific to adipose precursor cells of each depot and to evaluate each distinct adipose precursor population and its specific changes during senescence.

## DISTINCT NEW ADIPOSE PRECURSORS THAT EMERGE IN SAT AND VAT DURING AGING

Changes in certain adipose precursor populations during aging have recently been reported. A SAT-specific adipose precursor population in aging was discovered by Nguyen et al., named aging-dependent regulatory cells (ARCs).^84^ Comparisons of scRNA-seq of SVF cells from iSAT of 10-week-old (young), 48-week-old (aged), and 72-week-old (old) mice showed that ARCs arise only in SAT of aged and old mice and were undetectable in VAT of aged mice or mice on high-fat feeding. ARCs could also be detected in the SAT of aged but not young humans. ARCs had robust proliferative capacity and did not exhibit hallmarks of cellular senescence. ARCs arose from adipose precursors but exhibited impaired differentiation capacity, as isolated ARCs from mice were unable to differentiate into adipocytes. ARCs highly expressed inflammatory genes, and by secreting cytokines, such as CCL6, ARCs inhibited the proliferation and differentiation of neighboring adipose precursors *in vitro* and *in vivo*. Preadipocytes cultured in conditioned media from ARCs exhibited a significantly reduced adipogenic capacity, and 3T3-L1 cells transplanted into mice along with ARCs could not undergo adipocyte differentiation. The development of ARCs in SAT was shown to be driven by PU.1. The ectopic expression of PU.1 in 3T3-L1 cells resulted in an ARC-like phenotype with reduced adipogenic capacity and increased expression of inflammatory genes and chemokines. Conversely, knockdown of PU.1 in ARCs rescued adipogenic capacity. ARCs that appear in aging contribute to the aging-associated decrease of SAT.

More recently, another adipose precursor population that emerges in both SAT and VAT in aging mice was reported.^85^ Wang et al. showed that age-related fat accumulation was accompanied by adipogenesis. Compared to VAT from 6-month-old mice, 12-month-old mice had increased perilipin^+^ GFP^−^ cells, indicating high adipogenesis as well as increased adipocyte hypertrophy. By scRNA-seq of Lin^−^ SVF cells from VAT of 2.5- and 12-month-old mice, they identified a preadipocyte population that was age-enriched (CP-A) that developed from adipose precursors. CP-As displayed high proliferation and differentiation capacities, both *in vitro* and *in vivo*. Interestingly, the number of CP-As in VAT increased when mice were 9 months old, peaked at 12 months, and then sharply declined at 18 months, indicating that the generation of CP-As was an age-specific phenomenon. Leukemia inhibitory factor receptor was identified as a functional marker of CP-As indispensable for their adipogenic differentiation. Human CP-A population was identified in the peripancreatic VAT. The emergence of CP-As may explain VAT accumulation during early and middle aging. However, CP-As in SAT may be inhibited by SAT-specific ARCs, leading to a decrease in SAT during aging. It is likely that the senescent microenvironment promotes adipogenesis of adipose precursors, such as CP-As, in VAT during early aging, while this increase in adipogenesis is ablated with the emergence of PU.1^+^ ARCs in SAT.

**Figure 2.**
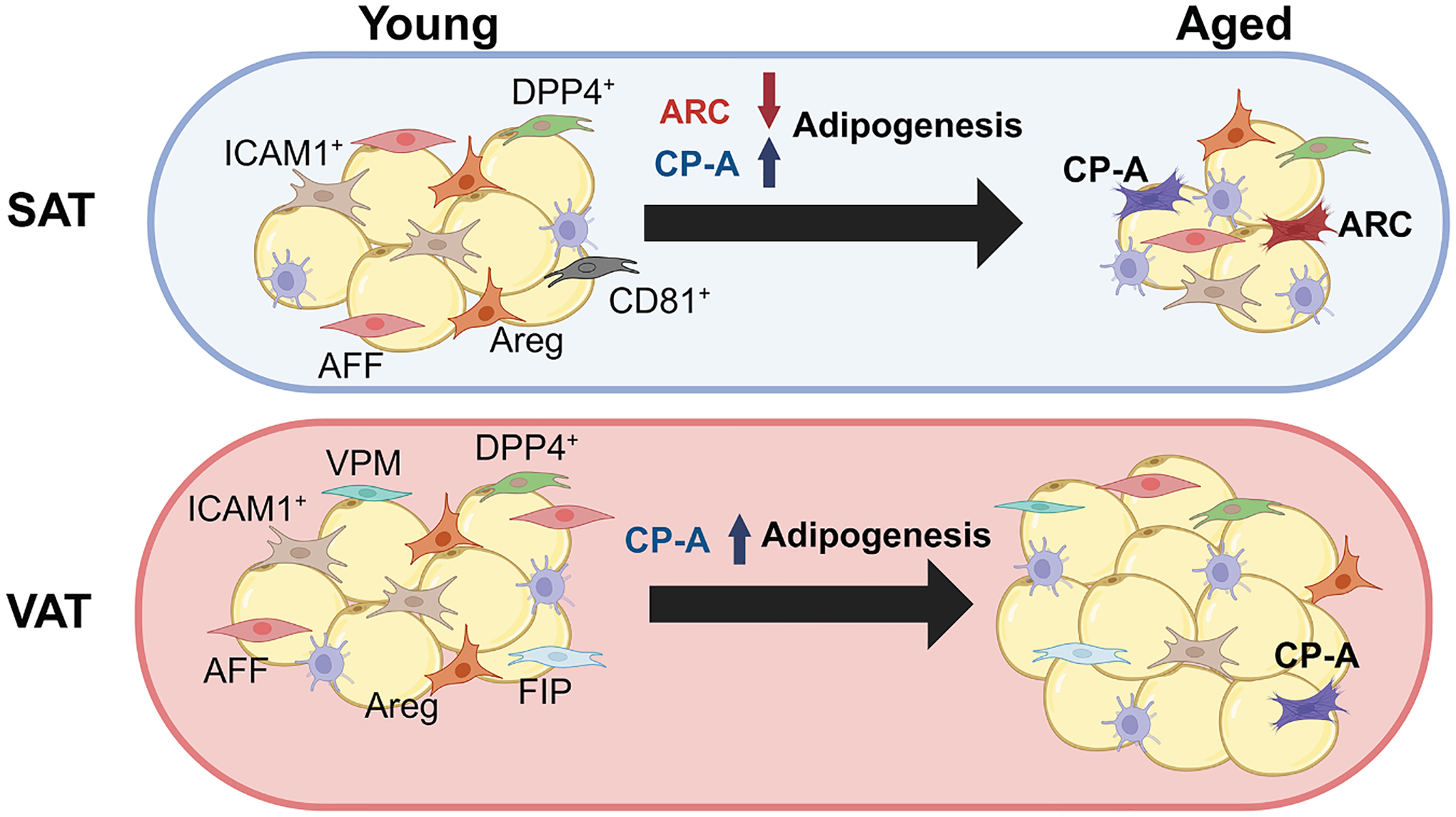
Distinct new adipose precursor populations emerging in SAT and VAT during aging SAT and VAT change in a depot-specific manner during aging. While VAT generally increases during aging, SAT is known to decrease significantly. During aging, new adipose precursor populations arise, notably a SAT-specific aging-dependent regulatory cell (ARC) population and an age-enriched committed preadipocyte (CP-A) population. ARCs have impaired differentiation capacity and inhibit the differentiation of neighboring preadipocytes. CP-As first increase in early aging, but decrease at later stages of aging while displaying high adipogenic potential. The emergence of CP-As may explain VAT accumulation during aging, but this effect may be inhibited in SAT by SAT-specific ARCs, resulting in a decrease in SAT during aging. While changes in other adipose precursor populations during aging require additional investigation, in general, adipose precursors as well as beige adipocytes decrease during aging.

The role of ARCs and CP-As in WAT during aging may play a key role in the differences observed in the distribution of SAT and VAT during aging. The emergence of CP-As in SAT and VAT during early and middle aging may play an adaptive role in compensating for the reduced adipogenesis of SAT, due to ARCs (Figure 2). Thus, ARCs may restrict the capacity of SAT to expand by inhibiting adipogenesis, leading to a positive systemic energy balance. Increased adipogenesis in VAT through CP-As may restore the energy balance to prevent ectopic triglyceride deposition. In fact, an increase in WAT mass through hyperplasia does not necessarily contribute negatively to age-related metabolic disorders and may prove beneficial by allowing the storage of excess energy and maintaining homeostatic energy balance. However, this beneficial effect may become detrimental during periods of unrestricted WAT expansion, leading to adipocyte hypertrophy and inflammation. The decline of the CP-A population in old age in VAT may also explain the positive correlation of metabolic disorders with age. The loss of CP-As in old age could decrease the ability of VAT to expand by hyperplasia, resulting in increased adipocyte hypertrophy and inflammation. Overall, the emergence of ARCs and CP-As may be crucial in the loss of protective SAT and increase in pathogenic VAT. What factors influence ARCs and CP-As to arise in aging require further investigation.

Aging-dependent changes are also predicted in other adipose precursor populations in addition to ARCs and CP-As, including mesenchymal stem cells, adipose progenitors, and preadipocytes.^84,121^ PanSci UMAP of SAT and VAT precursor populations reported by Zhang et al. displayed precursor populations in SAT and VAT diverging from other stromal populations in young mice.^121^ Precursor populations of each depot may possess distinct depot-specific qualities, giving changes in adipocyte populations observed during aging, and stromal populations may have a role in altering the development and function of emerging adipocytes. However, how different populations, such as the VAT-specific FIPs, are affected by aging has not been explored. Changes in the characteristics and the differentiation capacity of various adipose precursors during aging to bring distinct alterations in VAT and SAT require further investigation.

## DECLINE OF BEIGE ADIPOGENESIS IN SAT

A decrease in beiging, particularly in SAT, has been observed during aging in mice and humans.^112,125,144^ scRNA-seq of SAT adipocytes revealed that beige adipocytes undergo dramatic changes during aging. Beige adipocytes in mice during aging had increased expression of Cd9, fibro-inflammatory genes, and Npr3, an inhibitor of beige adipogenesis, and displayed impaired *de novo lipogenesis*.^145^ Pdgfrβ was previously shown to mark beige adipose precursors and is required for beige fat formation and IL-33 expression.^146^ Berry et al. showed an age-dependent upregulation in Pdgfrβ expression in mice, which downregulated IL-33 production by beige adipocytes. IL-33 was previously shown to activate type 2 innate lymphoid cells (ILC2s), which in turn support the proliferation of adipose precursors and their commitment to beige adipocyte lineage.^147^ However, Pdgfrβ deletion to upregulate IL-33 alone was not sufficient to fully restore beige adipogenesis,^146^ indicating that the decline in beige adipogenesis is driven by multiple factors, including dysfunction of ILC2 during aging.^148^ SAT adipocytes of aged mice and humans had decreased expression of PRDM16, a beige adipogenic factor, and loss of Prdm16 in aged mice reduced fibrosis and restored beige adipogenesis through the secretion of β-hydroxybutyrate.^149^ Loss of Prdm16 in SAT in aging could be due to increased expression of ubiquitin E3 ligase Cul2-Appbp2 for polyubiquitination and degradation of Prdm16.^150^ A recent study in mice suggested a decrease in estrogen levels with age, since treatment with estrogen (E2) ameliorated age-related defects in iWAT beiging.^151^ However, E2 treatment did not result in significant differences in fat content in females compared to males.

A reduction in beige adipogenesis may drive adipose tissue fibrosis and dysfunction. Rescuing beige adipocyte biogenesis may reduce adipose tissue fibrosis, improving adipogenesis and systemic glucose homeostasis. Indeed, it has been shown that repression of adipose tissue fibrosis through PRDM16-GTF2IRD1 complex in SAT of mice represses adipose tissue fibrosis and improves systemic glucose homeostasis.^152^ Overall, while adipose tissue fibrosis is observed in both SAT and VAT depots in aging, the decline of beige adipogenesis in aging may impact SAT in a depot-specific manner, increasing fibrosis and reducing the protective functions of SAT. However, the molecular mechanisms driving this change in beige precursors are poorly understood and require further investigation. Senescence could be a driver of reduced beige adipogenesis, as Berry et al. demonstrated reversing of cellular aging in beige precursor cells by targeting the senescent pathway p38/MAPK-p16^Ink4a^ in SAT of aged mice and human beige progenitor cells.^112^ However, the molecular differences of beige adipocytes between rodents and humans could limit the direct applicability of these findings to human therapeutic strategies.

## CONCLUSION

**Figure 3.**
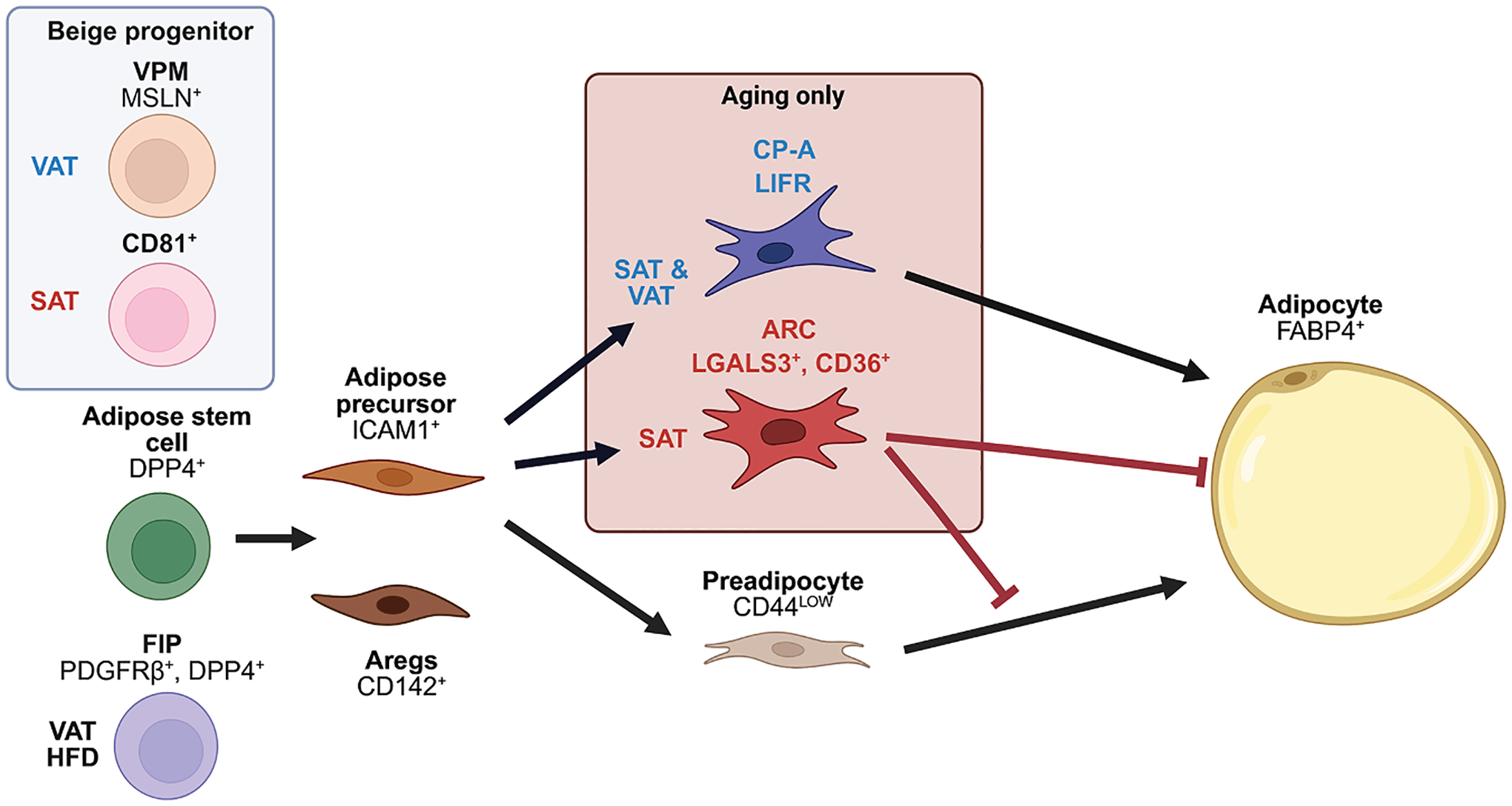
Adipose precursor populations in development and in aging Adipose precursor cells are heterogeneous and represent different stages in the differentiation timeline. A DPP4^+^ precursor population, considered to be stem-like, exists in both SAT and VAT. FIPs are identified as a VAT-specific inflammatory adipose stem cell population that increases upon high-fat feeding. Depot-specific beige adipocyte progenitors have also been identified. CD81^+^ beige progenitors are reported to give rise to beige adipocytes specifically in SAT. VPMs are identified as beige progenitors in VAT with high mitochondrial gene expression. ICAM1^+^ precursors and CD142^+^ Aregs are more committed adipose precursor populations found in both SAT and VAT. Highly committed preadipocytes were identified by having a low expression of CD44, compared to less committed CD44^high^ adipose precursor populations. During aging, two distinct adipose precursor populations emerge. An adipose lineage ARC population emerges in a SAT-specific manner; ARCs resist adipogenesis and inhibit the adipogenesis of neighboring adipose precursors by secretion of chemokines, such as CCL6. Another adipose lineage CP-A population emerges in both SAT and VAT and displays a high differentiation capacity. An adipose lineage ARC population emerges in a SAT-specific manner; ARCs resist adipogenesis and inhibit the adipogenesis of neighboring adipose precursors by secretion of chemokines, such as CCL6. Another adipose lineage CP-A population emerges in both SAT and VAT and displays high differentiation capacity.

WAT is a dynamic organ with high plasticity to maintain energy homeostasis. The two major WAT depots, subcutaneous and visceral, are differentially correlated with metabolic disease risk. While VAT is associated with pathological conditions, such as insulin resistance and cardiovascular disease, SAT is known to be protective against these diseases. During aging, adipose tissue becomes dysfunctional and undergoes changes in mass, distribution, and cellular composition. Overall, VAT mass tends to increase, whereas SAT mass decreases during aging. Accumulation of VAT in the presence of other aging pathologies further contributes to the acceleration of aging and shortening of the health span, while loss in SAT is linked to increased insulin resistance and risk of diabetes. Lineage tracing and single-cell analyses have been used to understand the complexities and differences between SAT and VAT in developmental origins and adipose precursors, as well as changes in adipose precursor populations during aging (Figure 3). However, how these populations emerge and their interactions with other populations in adipose tissue are not well understood.

Dysfunctional adipose tissue in aging contributes to impairment in metabolism and metabolic disease, such as ectopic fat deposition, visceral obesity, and insulin resistance. A better understanding of the various depot-specific adipose precursor populations and their interactions may provide targets to modify body fat distribution, control inflammation, and prevent the onset of age-related metabolic disease. Targeting specific precursor cell populations, such as ARCs, may improve adipose function by rescuing adipogenic potential and thus metabolic health. New insights into the identities and regulations of adipose precursor populations may reveal novel drug targets to promote metabolically beneficial tissue remodeling. Targeting adipose precursor fate and supporting their adipogenesis may combat adipocyte hypertrophy, inflammation, and fibrosis observed in metabolic disease and aging.

## Figures and Tables

**Table 1.** Distinct adipose precursor populations identified in SAT and VAT

Population name	Anatomical depot	Species	Population markers	Characteristics	Enriched pathways	Reference
DPP4^+^	SAT and VAT	Mouse and human	*CD34*, *PDGFR*α, *LY6A*, and CD90	Multipotent progenitors, highly proliferative, and refractory to adipogenesis	TGFβ, WNT	Merrick et al.^82^
ICAM1^+^	SAT and VAT	Mouse and human	CD54,^82^ COL4A2,^83^ AOC3,^84^ and CD44^90^	Committed preadipocyte and adipogenic	Adipocyte differentiation	Merrick et al.^82^; Burl et al.^83^; Nguyen et al.^84^; and Chen et al.^90^
CD142^+^ (Areg)	SAT and VAT	Mouse, human	ABCG1,^81^ *CLEC11a*,^82^ *RAMP1*, and *AOX3*^84^	Refractory to adipogenesis and inhibits adipogenesis; adipogenic	Hedgehog signaling	Schwalie et al.^81^; Merrick et al.^82^; and Nguyen et al.^91^
FIP	VAT (high-fat feeding)	Mouse	DPP4, LY6C, and PDGFRβ	Increases during HFD; refractory to adipogenesis and exerts anti-adipogenic effects	Inflammatory cytokines (IL-6, CXCL2, and CXCL10)	Hepler et al.^86^
CD81 beige progenitor	SAT	Mouse and human	PDGFRα, *LY6A*, and CD81	Highly proliferative and beige adipocyte precursor	Cell growth, actin filament processes, apoptotic signaling, cell cycle, and wound healing	Oguri et al.^92^
VPM	VAT	Human	*MSLN*, *UPK3B*, and *WT1*	Beige adipocyte precursor in VAT	Mitochondrial gene expression	Vijay et al.^87^
ARC	SAT (aging only)	Mouse and human	CD36, LGALS3, and PU.1	Increases during aging, is refractory to adipogenesis, and inhibits adipogenesis	Inflammatory cytokines (CCL6)	Nguyen et al.^84^
CP-A	SAT and VAT (aging only)	Mouse and human	LIFR	Increases during aging; highly proliferative and adipogenic	Adipocyte differentiation	Wang et al.^85^

## References

[1] Shook BA, Wasko RR, Mano O, Rutenberg-Schoenberg M, Rudolph MC, Zirak B, Rivera-Gonzalez GC, López-Giráldez F, Zarini S, Rezza A, (2020). Dermal Adipocyte Lipolysis and Myofibroblast Conversion Are Required for Efficient Skin Repair. Cell Stem Cell 26, 880–895.e6. 32302523 10.1016/j.stem.2020.03.013 PMC7853423

[2] Festa E, Fretz J, Berry R, Schmidt B, Rodeheffer M, Horowitz M, and Horsley V (2011). Adipocyte lineage cells contribute to the skin stem cell niche to drive hair cycling. Cell 146, 761–771. 21884937 10.1016/j.cell.2011.07.019 PMC3298746

[3] Zhong L, Yao L, Tower RJ, Wei Y, Miao Z, Park J, Shrestha R, Wang L, Yu W, Holdreith N, (2020). Single cell transcriptomics identifies a unique adipose lineage cell population that regulates bone marrow environment. eLife 9, e54695. 32286228 10.7554/eLife.54695 PMC7220380

[4] Suchacki KJ, Tavares AAS, Mattiucci D, Scheller EL, Papanastasiou G, Gray C, Sinton MC, Ramage LE, McDougald WA, Lovdel A, (2020). Bone marrow adipose tissue is a unique adipose subtype with distinct roles in glucose homeostasis. Nat. Commun 11, 3097. 32555194 10.1038/s41467-020-16878-2 PMC7303125

[5] Stern JH, Rutkowski JM, and Scherer PE (2016). Adiponectin, Leptin, and Fatty Acids in the Maintenance of Metabolic Homeostasis through Adipose Tissue Crosstalk. Cell Metab 23, 770–784. 27166942 10.1016/j.cmet.2016.04.011 PMC4864949

[6] Brown RJ, Araujo-Vilar D, Cheung PT, Dunger D, Garg A, Jack M, Mungai L, Oral EA, Patni N, Rother KI, (2016). The Diagnosis and Management of Lipodystrophy Syndromes: A Multi-Society Practice Guideline. J. Clin. Endocrinol. Metab 101, 4500–4511. 27710244 10.1210/jc.2016-2466 PMC5155679

[7] Pendse AA, Johnson LA, Tsai YS, and Maeda N (2010). Pparg-P465L mutation worsens hyperglycemia in Ins2-Akita female mice via adipose-specific insulin resistance and storage dysfunction. Diabetes 59, 2890–2897. 20724579 10.2337/db10-0673 PMC2963548

[8] Barroso I, Gurnell M, Crowley VE, Agostini M, Schwabe JW, Soos MA, Maslen GL, Williams TD, Lewis H, Schafer AJ, (1999). Dominant negative mutations in human PPARgamma associated with severe insulin resistance, diabetes mellitus and hypertension. Nature 402, 880–883. 10622252 10.1038/47254

[9] Pajed L, Taschler U, Tilp A, Hofer P, Kotzbeck P, Kolleritsch S, Radner FPW, Pototschnig I, Wagner C, Schratter M, (2021). Advanced lipodystrophy reverses fatty liver in mice lacking adipocyte hormone-sensitive lipase. Commun. Biol 4, 323. 33692445 10.1038/s42003-021-01858-z PMC7946939

[10] Lindroos J, Husa J, Mitterer G, Haschemi A, Rauscher S, Haas R, Gröger M, Loewe R, Kohrgruber N, Schrögendorfer KF, (2013). Human but not mouse adipogenesis is critically dependent on LMO3. Cell Metab 18, 62–74. 23823477 10.1016/j.cmet.2013.05.020 PMC3701325

[11] Duncan RE, Ahmadian M, Jaworski K, Sarkadi-Nagy E, and Sul HS (2007). Regulation of lipolysis in adipocytes. Annu. Rev. Nutr 27, 79–101. 17313320 10.1146/annurev.nutr.27.061406.093734 PMC2885771

[12] Ding H, Zheng S, Garcia-Ruiz D, Hou D, Wei Z, Liao Z, Li L, Zhang Y, Han X, Zen K, (2016). Fasting induces a subcutaneous-to-visceral fat switch mediated by microRNA-149-3p and suppression of PRDM16. Nat. Commun 7, 11533. 27240637 10.1038/ncomms11533 PMC4895052

[13] Merlotti C, Ceriani V, Morabito A, and Pontiroli AE (2017). Subcutaneous fat loss is greater than visceral fat loss with diet and exercise, weight-loss promoting drugs and bariatric surgery: a critical review and meta-analysis. Int. J. Obes 41, 672–682. 10.1038/ijo.2017.31 28148928

[14] Han J, Wang Y, Qiu Y, Sun D, Liu Y, Li Z, Zhou B, Zhang H, Xiao Y, Wu G, and Ding Q (2022). Single-cell sequencing unveils key contributions of immune cell populations in cancer-associated adipose wasting. Cell Discov 8, 122. 36376273 10.1038/s41421-022-00466-3 PMC9663454

[15] Krapić M, Kavazović I, Mikašinović S, Mladenić K, Krstanović F, Seyhan G, Helmrath S, Camerini E, Brizić I, Peters FS, (2025). NK cell-derived IFNγ mobilizes free fatty acids from adipose tissue to promote early B cell activation during viral infection. Nat. Metab 7, 985–1003. 40217117 10.1038/s42255-025-01273-2

[16] Jackson-Jones LH, Smith P, Portman JR, Magalhaes MS, Mylonas KJ, Vermeren MM, Nixon M, Henderson BEP, Dobie R, Vermeren S, (2020). Stromal Cells Covering Omental Fat-Associated Lymphoid Clusters Trigger Formation of Neutrophil Aggregates to Capture Peritoneal Contaminants. Immunity 52, 700–715.e6. 32294409 10.1016/j.immuni.2020.03.011 PMC7156918

[17] Cipolletta D, Feuerer M, Li A, Kamei N, Lee J, Shoelson SE, Benoist C, and Mathis D (2012). PPAR-γ is a major driver of the accumulation and phenotype of adipose tissue Treg cells. Nature 486, 549–553. 22722857 10.1038/nature11132 PMC3387339

[18] Kajimura S, Spiegelman BM, and Seale P (2015). Brown and Beige Fat: Physiological Roles beyond Heat Generation. Cell Metab 22, 546–559. 26445512 10.1016/j.cmet.2015.09.007 PMC4613812

[19] Shinoda K, Luijten IHN, Hasegawa Y, Hong H, Sonne SB, Kim M, Xue R, Chondronikola M, Cypess AM, Tseng YH, (2015). Genetic and functional characterization of clonally derived adult human brown adipocytes. Nat. Med 21, 389–394. 25774848 10.1038/nm.3819 PMC4427356

[20] Lee P, Swarbrick MM, Zhao JT, and Ho KKY (2011). Inducible brown adipogenesis of supraclavicular fat in adult humans. Endocrinology 152, 3597–3602. 21791556 10.1210/en.2011-1349

[21] Jespersen NZ, Larsen TJ, Peijs L, Daugaard S, Homøe P, Loft A, de Jong J, Mathur N, Cannon B, Nedergaard J, (2013). A classical brown adipose tissue mRNA signature partly overlaps with brite in the supraclavicular region of adult humans. Cell Metab 17, 798–805. 23663743 10.1016/j.cmet.2013.04.011

[22] Cypess AM, White AP, Vernochet C, Schulz TJ, Xue R, Sass CA, Huang TL, Roberts-Toler C, Weiner LS, Sze C, (2013). Anatomical localization, gene expression profiling and functional characterization of adult human neck brown fat. Nat. Med 19, 635–639. 23603815 10.1038/nm.3112 PMC3650129

[23] de Jong JMA, Sun W, Pires ND, Frontini A, Balaz M, Jespersen NZ, Feizi A, Petrovic K, Fischer AW, Bokhari MH, (2019). Human brown adipose tissue is phenocopied by classical brown adipose tissue in physiologically humanized mice. Nat. Metab 1, 830–843. 32694768 10.1038/s42255-019-0101-4

[24] Cero C, Lea HJ, Zhu KY, Shamsi F, Tseng YH, and Cypess AM (2021). β3-Adrenergic receptors regulate human brown/beige adipocyte lipolysis and thermogenesis. JCI Insight 6, e139160. 34100382 10.1172/jci.insight.139160 PMC8262278

[25] Wernstedt Asterholm I, Tao C, Morley TS, Wang QA, Delgado-Lopez F, Wang ZV, and Scherer PE (2014). Adipocyte Inflammation Is Essential for Healthy Adipose Tissue Expansion and Remodeling. Cell Metab 20, 103–118. 24930973 10.1016/j.cmet.2014.05.005 PMC4079756

[26] Cao Y (2007). Angiogenesis modulates adipogenesis and obesity. J. Clin. Investig 117, 2362–2368. 17786229 10.1172/JCI32239 PMC1963348

[27] Shen H, Huang X, Zhao Y, Wu D, Xue K, Yao J, Wang Y, Tang N, and Qiu Y (2022). The Hippo pathway links adipocyte plasticity to adipose tissue fibrosis. Nat. Commun 13, 6030. 36229481 10.1038/s41467-022-33800-0 PMC9562301

[28] Vandanmagsar B, Youm YH, Ravussin A, Galgani JE, Stadler K, Mynatt RL, Ravussin E, Stephens JM, and Dixit VD (2011). The NLRP3 inflammasome instigates obesity-induced inflammation and insulin resistance. Nat. Med 17, 179–188. 21217695 10.1038/nm.2279 PMC3076025

[29] Marcelin G, Ferreira A, Liu Y, Atlan M, Aron-Wisnewsky J, Pelloux V, Botbol Y, Ambrosini M, Fradet V, Rouault C, (2017). A PDGFRα-Mediated Switch toward CD9high Adipocyte Progenitors Controls Obesity-Induced Adipose Tissue Fibrosis. Cell Metab 25, 673–685. 28215843 10.1016/j.cmet.2017.01.010

[30] Zhang L, Cai X, Wu X, Jing Z, Zhao Y, Yao Y, and Boström KI (2025). Regulating the cell differentiation trajectory of progenitor cells in adipose tissue fibrosis. Mol. Metab 100, 102231. 40780446 10.1016/j.molmet.2025.102231 PMC12396487

[31] Wang L, Pydi SP, Zhu L, Barella LF, Cui Y, Gavrilova O, Bence KK, Vernochet C, and Wess J (2020). Adipocyte Gi signaling is essential for maintaining whole-body glucose homeostasis and insulin sensitivity. Nat. Commun 11, 2995. 32532984 10.1038/s41467-020-16756-x PMC7293267

[32] Li H, Wu G, Fang Q, Zhang M, Hui X, Sheng B, Wu L, Bao Y, Li P, Xu A, and Jia W (2018). Fibroblast growth factor 21 increases insulin sensitivity through specific expansion of subcutaneous fat. Nat. Commun 9, 272. 29348470 10.1038/s41467-017-02677-9 PMC5773530

[33] Tran TT, Yamamoto Y, Gesta S, and Kahn CR (2008). Beneficial effects of subcutaneous fat transplantation on metabolism. Cell Metab 7, 410–420. 18460332 10.1016/j.cmet.2008.04.004 PMC3204870

[34] Kuang J, Zhang Y, Liu Q, Shen J, Pu S, Cheng S, Chen L, Li H, Wu T, Li R, (2017). Fat-Specific Sirt6 Ablation Sensitizes Mice to High-Fat Diet–Induced Obesity and Insulin Resistance by Inhibiting Lipolysis. Diabetes 66, 1159–1171. 28250020 10.2337/db16-1225

[35] Furukawa S, Fujita T, Shimabukuro M, Iwaki M, Yamada Y, Nakajima Y, Nakayama O, Makishima M, Matsuda M, and Shimomura I (2004). Increased oxidative stress in obesity and its impact on metabolic syndrome. J. Clin. Investig 114, 1752–1761. 15599400 10.1172/JCI21625 PMC535065

[36] Verboven K, Wouters K, Gaens K, Hansen D, Bijnen M, Wetzels S, Stehouwer CD, Goossens GH, Schalkwijk CG, Blaak EE, and Jocken JW (2018). Abdominal subcutaneous and visceral adipocyte size, lipolysis and inflammation relate to insulin resistance in male obese humans. Sci. Rep 8, 4677. 29549282 10.1038/s41598-018-22962-x PMC5856747

[37] Feuerer M, Herrero L, Cipolletta D, Naaz A, Wong J, Nayer A, Lee J, Goldfine AB, Benoist C, Shoelson S, and Mathis D (2009). Lean, but not obese, fat is enriched for a unique population of regulatory T cells that affect metabolic parameters. Nat. Med 15, 930–939. 19633656 10.1038/nm.2002 PMC3115752

[38] Jeffery E, Church CD, Holtrup B, Colman L, and Rodeheffer MS (2015). Rapid depot-specific activation of adipocyte precursor cells at the onset of obesity. Nat. Cell Biol 17, 376–385. 25730471 10.1038/ncb3122 PMC4380653

[39] Smas CM, and Sul HS (1993). Pref-1, a protein containing EGF-like repeats, inhibits adipocyte differentiation. Cell 73, 725–734. 8500166 10.1016/0092-8674(93)90252-l

[40] Lee K, Villena JA, Moon YS, Kim KH, Lee S, Kang C, and Sul HS (2003). Inhibition of adipogenesis and development of glucose intolerance by soluble preadipocyte factor-1 (Pref-1). J. Clin. Investig 111, 453–461. 12588883 10.1172/JCI15924 PMC151920

[41] Rodeheffer MS, Birsoy K, and Friedman JM (2008). Identification of white adipocyte progenitor cells in vivo. Cell 135, 240–249. 18835024 10.1016/j.cell.2008.09.036

[42] Hudak CS, Gulyaeva O, Wang Y, Park SM, Lee L, Kang C, and Sul HS (2014). Pref-1 marks very early mesenchymal precursors required for adipose tissue development and expansion. Cell Rep 8, 678–687. 25088414 10.1016/j.celrep.2014.06.060 PMC4138044

[43] Berry R, and Rodeheffer MS (2013). Characterization of the adipocyte cellular lineage in vivo. Nat. Cell Biol 15, 302–308. 23434825 10.1038/ncb2696 PMC3721064

[44] Gulyaeva O, Nguyen H, Sambeat A, Heydari K, and Sul HS (2018). Sox9-Meis1 Inactivation Is Required for Adipogenesis, Advancing Pref-1(+) to PDGFRalpha(+) Cells. Cell Rep 25, 1002–1017.e4. 30355480 10.1016/j.celrep.2018.09.086 PMC6903418

[45] Wang Y, and Sul HS (2009). Pref-1 regulates mesenchymal cell commitment and differentiation through Sox9. Cell Metab 9, 287–302. 19254573 10.1016/j.cmet.2009.01.013 PMC2673480

[46] Ross SE, Hemati N, Longo KA, Bennett CN, Lucas PC, Erickson RL, and MacDougald OA (2000). Inhibition of adipogenesis by Wnt signaling. Science 289, 950–953. 10937998 10.1126/science.289.5481.950

[47] Spalding KL, Arner E, Westermark PO, Bernard S, Buchholz BA, Bergmann O, Blomqvist L, Hoffstedt J, Näslund E, Britton T, (2008). Dynamics of fat cell turnover in humans. Nature 453, 783–787. 18454136 10.1038/nature06902

[48] Wang QA, Tao C, Gupta RK, and Scherer PE (2013). Tracking adipogenesis during white adipose tissue development, expansion and regeneration. Nat. Med 19, 1338–1344. 23995282 10.1038/nm.3324 PMC4075943

[49] Sun K, Kusminski CM, and Scherer PE (2011). Adipose tissue remodeling and obesity. J. Clin. Investig 121, 2094–2101. 21633177 10.1172/JCI45887 PMC3104761

[50] Engler AJ, Sen S, Sweeney HL, and Discher DE (2006). Matrix elasticity directs stem cell lineage specification. Cell 126, 677–689. 16923388 10.1016/j.cell.2006.06.044

[51] Abuhattum S, Kotzbeck P, Schlüßler R, Harger A, Ariza de Schellenberger A, Kim K, Escolano JC, Müller T, Braun J, Wabitsch M, (2022). Adipose cells and tissues soften with lipid accumulation while in diabetes adipose tissue stiffens. Sci. Rep 12, 10325. 35725987 10.1038/s41598-022-13324-9 PMC9209483

[52] Gonzalez Porras MA, Stojkova K, Vaicik MK, Pelowe A, Goddi A, Carmona A, Long B, Qutub AA, Gonzalez A, Cohen RN, and Brey EM (2021). Integrins and extracellular matrix proteins modulate adipocyte thermogenic capacity. Sci. Rep 11, 5442. 33686208 10.1038/s41598-021-84828-z PMC7940610

[53] Wernstedt Asterholm I, Tao C, Morley TS, Wang QA, Delgado-Lopez F, Wang ZV, and Scherer PE (2014). Adipocyte inflammation is essential for healthy adipose tissue expansion and remodeling. Cell Metab 20, 103–118. 24930973 10.1016/j.cmet.2014.05.005 PMC4079756

[54] Bapat SP, Myoung Suh J, Fang S, Liu S, Zhang Y, Cheng A, Zhou C, Liang Y, LeBlanc M, Liddle C, (2015). Depletion of fat-resident Treg cells prevents age-associated insulin resistance. Nature 528, 137–141. 26580014 10.1038/nature16151 PMC4670283

[55] Traktuev DO, Prater DN, Merfeld-Clauss S, Sanjeevaiah AR, Saadatzadeh MR, Murphy M, Johnstone BH, Ingram DA, and March KL (2009). Robust functional vascular network formation in vivo by cooperation of adipose progenitor and endothelial cells. Circ. Res 104, 1410–1420. 19443841 10.1161/CIRCRESAHA.108.190926

[56] Traktuev DO, Merfeld-Clauss S, Li J, Kolonin M, Arap W, Pasqualini R, Johnstone BH, and March KL (2008). A Population of Multipotent CD34-Positive Adipose Stromal Cells Share Pericyte and Mesenchymal Surface Markers, Reside in a Periendothelial Location, and Stabilize Endothelial Networks. Circ. Res 102, 77–85. 17967785 10.1161/CIRCRESAHA.107.159475

[57] Isakson P, Hammarstedt A, Gustafson B, and Smith U (2009). Impaired preadipocyte differentiation in human abdominal obesity: role of Wnt, tumor necrosis factor-alpha, and inflammation. Diabetes 58, 1550–1557. 19351711 10.2337/db08-1770 PMC2699851

[58] Charrière G, Cousin B, Arnaud E, André M, Bacou F, Penicaud L, and Casteilla L (2003). Preadipocyte conversion to macrophage. Evidence of plasticity. J. Biol. Chem 278, 9850–9855. 12519759 10.1074/jbc.M210811200

[59] Poissonnet CM, Burdi AR, and Garn SM (1984). The chronology of adipose tissue appearance and distribution in the human fetus. Early Hum. Dev 10, 1–11. 6499712 10.1016/0378-3782(84)90106-3

[60] Tchkonia T, Lenburg M, Thomou T, Giorgadze N, Frampton G, Pirtskhalava T, Cartwright A, Cartwright M, Flanagan J, Karagiannides I, (2007). Identification of depot-specific human fat cell progenitors through distinct expression profiles and developmental gene patterns. Am. J. Physiol. Endocrinol. Metab 292, E298–E307. 16985259 10.1152/ajpendo.00202.2006

[61] Gesta S, Blüher M, Yamamoto Y, Norris AW, Berndt J, Kralisch S, Boucher J, Lewis C, and Kahn CR (2006). Evidence for a role of developmental genes in the origin of obesity and body fat distribution. Proc. Natl. Acad. Sci. USA 103, 6676–6681. 16617105 10.1073/pnas.0601752103 PMC1458940

[62] Chau YY, Bandiera R, Serrels A, Martínez-Estrada OM, Qing W, Lee M, Slight J, Thornburn A, Berry R, McHaffie S, (2014). Visceral and subcutaneous fat have different origins and evidence supports a mesothelial source. Nat. Cell Biol 16, 367–375. 24609269 10.1038/ncb2922 PMC4060514

[63] Westcott GP, Emont MP, Li J, Jacobs C, Tsai L, and Rosen ED (2021). Mesothelial cells are not a source of adipocytes in mice. Cell Rep 36, 109388. 34260927 10.1016/j.celrep.2021.109388 PMC8317472

[64] Liu Q, Li C, Deng B, Gao P, Wang L, Li Y, Shiri M, Alkaifi F, Zhao J, Stephens JM, (2023). Tcf21 marks visceral adipose mesenchymal progenitors and functions as a rate-limiting factor during visceral adipose tissue development. Cell Rep 42, 112166. 36857185 10.1016/j.celrep.2023.112166 PMC10208561

[65] Krueger KC, Costa MJ, Du H, and Feldman BJ (2014). Characterization of Cre recombinase activity for in vivo targeting of adipocyte precursor cells. Stem Cell Rep 3, 1147–1158. 10.1016/j.stemcr.2014.10.009 PMC4264060 25458893

[66] Sanchez-Gurmaches J, Hsiao WY, and Guertin DA (2015). Highly selective in vivo labeling of subcutaneous white adipocyte precursors with Prx1-Cre. Stem Cell Rep 4, 541–550. 10.1016/j.stemcr.2015.02.008 PMC4400610 25801508

[67] Sanchez-Gurmaches J, and Guertin DA (2014). Adipocytes arise from multiple lineages that are heterogeneously and dynamically distributed. Nat. Commun 5, 4099. 24942009 10.1038/ncomms5099 PMC4066194

[68] Gupta RK, Arany Z, Seale P, Mepani RJ, Ye L, Conroe HM, Roby YA, Kulaga H, Reed RR, and Spiegelman BM (2010). Transcriptional control of preadipocyte determination by Zfp423. Nature 464, 619–623. 20200519 10.1038/nature08816 PMC2845731

[69] Tran KV, Gealekman O, Frontini A, Zingaretti MC, Morroni M, Giordano A, Smorlesi A, Perugini J, De Matteis R, Sbarbati A, (2012). The vascular endothelium of the adipose tissue gives rise to both white and brown fat cells. Cell Metab 15, 222–229. 22326223 10.1016/j.cmet.2012.01.008 PMC3278718

[70] Takashima Y, Era T, Nakao K, Kondo S, Kasuga M, Smith AG, and Nishikawa SI (2007). Neuroepithelial cells supply an initial transient wave of MSC differentiation. Cell 129, 1377–1388. 17604725 10.1016/j.cell.2007.04.028

[71] Billon N, Iannarelli P, Monteiro MC, Glavieux-Pardanaud C, Richardson WD, Kessaris N, Dani C, and Dupin E (2007). The generation of adipocytes by the neural crest. Development 134, 2283–2292. 17507398 10.1242/dev.002642 PMC6334830

[72] Long JZ, Svensson KJ, Tsai L, Zeng X, Roh HC, Kong X, Rao RR, Lou J, Lokurkar I, Baur W, (2014). A smooth muscle-like origin for beige adipocytes. Cell Metab 19, 810–820. 24709624 10.1016/j.cmet.2014.03.025 PMC4052772

[73] Berry DC, Jiang Y, and Graff JM (2016). Mouse strains to study cold-inducible beige progenitors and beige adipocyte formation and function. Nat. Commun 7, 10184. 26729601 10.1038/ncomms10184 PMC4728429

[74] Yamamoto Y, Gesta S, Lee KY, Tran TT, Saadatirad P, and Kahn CR (2010). Adipose depots possess unique developmental gene signatures. Obesity 18, 872–878. 20111017 10.1038/oby.2009.512 PMC4377838

[75] Macotela Y, Boucher J, Tran TT, and Kahn CR (2009). Sex and depot differences in adipocyte insulin sensitivity and glucose metabolism. Diabetes 58, 803–812. 19136652 10.2337/db08-1054 PMC2661589

[76] Macotela Y, Emanuelli B, Mori MA, Gesta S, Schulz TJ, Tseng YH, and Kahn CR (2012). Intrinsic differences in adipocyte precursor cells from different white fat depots. Diabetes 61, 1691–1699. 22596050 10.2337/db11-1753 PMC3379665

[77] Tchkonia T, Giorgadze N, Pirtskhalava T, Thomou T, DePonte M, Koo A, Forse RA, Chinnappan D, Martin-Ruiz C, von Zglinicki T, and Kirkland JL (2006). Fat depot-specific characteristics are retained in strains derived from single human preadipocytes. Diabetes 55, 2571–2578. 16936206 10.2337/db06-0540

[78] Meissburger B, Perdikari A, Moest H, Müller S, Geiger M, and Wolfrum C (2016). Regulation of adipogenesis by paracrine factors from adipose stromal-vascular fraction - a link to fat depot-specific differences. Biochim. Biophys. Acta 1861, 1121–1131. 27317982 10.1016/j.bbalip.2016.06.010

[79] Kim SM, Lun M, Wang M, Senyo SE, Guillermier C, Patwari P, and Steinhauser ML (2014). Loss of white adipose hyperplastic potential is associated with enhanced susceptibility to insulin resistance. Cell Metab 20, 1049–1058. 25456741 10.1016/j.cmet.2014.10.010 PMC4715375

[80] Petrus P, Mejhert N, Corrales P, Lecoutre S, Li Q, Maldonado E, Kulyté A, Lopez Y, Campbell M, Acosta JR, (2018). Transforming Growth Factor-β3 Regulates Adipocyte Number in Subcutaneous White Adipose Tissue. Cell Rep 25, 551–560.e5. 30332637 10.1016/j.celrep.2018.09.069

[81] Schwalie PC, Dong H, Zachara M, Russeil J, Alpern D, Akchiche N, Caprara C, Sun W, Schlaudraff KU, Soldati G, (2018). A stromal cell population that inhibits adipogenesis in mammalian fat depots. Nature 559, 103–108. 29925944 10.1038/s41586-018-0226-8

[82] Merrick D, Sakers A, Irgebay Z, Okada C, Calvert C, Morley MP, Percec I, and Seale P (2019). Identification of a mesenchymal progenitor cell hierarchy in adipose tissue. Science 364, eaav2501. 31023895 10.1126/science.aav2501 PMC6816238

[83] Burl RB, Ramseyer VD, Rondini EA, Pique-Regi R, Lee YH, and Granneman JG (2018). Deconstructing Adipogenesis Induced by beta3-Adrenergic Receptor Activation with Single-Cell Expression Profiling. Cell Metab 28, 300–309.e4. 29937373 10.1016/j.cmet.2018.05.025 PMC6082711

[84] Nguyen HP, Lin F, Yi D, Xie Y, Dinh J, Xue P, and Sul HS (2021). Aging-dependent regulatory cells emerge in subcutaneous fat to inhibit adipogenesis. Dev. Cell 56, 1437–1451.e3. 33878347 10.1016/j.devcel.2021.03.026 PMC8137669

[85] Wang G, Li G, Song A, Zhao Y, Yu J, Wang Y, Dai W, Salas M, Qin H, Medrano L, (2025). Distinct adipose progenitor cells emerging with age drive active adipogenesis. Science 388, eadj0430. 40273250 10.1126/science.adj0430 PMC12445215

[86] Hepler C, Shan B, Zhang Q, Henry GH, Shao M, Vishvanath L, Ghaben AL, Mobley AB, Strand D, Hon GC, and Gupta RK (2018). Identification of functionally distinct fibro-inflammatory and adipogenic stromal subpopulations in visceral adipose tissue of adult mice. eLife 7, e39636. 30265241 10.7554/eLife.39636 PMC6167054

[87] Vijay J, Gauthier MF, Biswell RL, Louiselle DA, Johnston JJ, Cheung WA, Belden B, Pramatarova A, Biertho L, Gibson M, (2020). Single-cell analysis of human adipose tissue identifies depot and disease specific cell types. Nat. Metab 2, 97–109. 32066997 10.1038/s42255-019-0152-6 PMC7025882

[88] Emont MP, Jacobs C, Essene AL, Pant D, Tenen D, Colleluori G, Di Vincenzo A, Jørgensen AM, Dashti H, Stefek A, (2022). A single-cell atlas of human and mouse white adipose tissue. Nature 603, 926–933. 35296864 10.1038/s41586-022-04518-2 PMC9504827

[89] Massier L, Jalkanen J, Elmastas M, Zhong J, Wang T, Nono Nankam PA, Frendo-Cumbo S, Bäckdahl J, Subramanian N, Sekine T, (2023). An integrated single cell and spatial transcriptomic map of human white adipose tissue. Nat. Commun 14, 1438. 36922516 10.1038/s41467-023-36983-2 PMC10017705

[90] Chen M, Kim S, Li L, Chattopadhyay S, Rando TA, and Feldman BJ (2023). Identification of an adipose tissue-resident pro-preadipocyte population. Cell Rep 42, 112440. 37119138 10.1016/j.celrep.2023.112440 PMC10370484

[91] Birnbaum RY, Clowney EJ, Agamy O, Kim MJ, Zhao J, Yamanaka T, Pappalardo Z, Clarke SL, Wenger AM, Nguyen L, (2012). Coding exons function as tissue-specific enhancers of nearby genes. Genome Res 22, 1059–1068. 22442009 10.1101/gr.133546.111 PMC3371700

[92] Oguri Y, Shinoda K, Kim H, Alba DL, Bolus WR, Wang Q, Brown Z, Pradhan RN, Tajima K, Yoneshiro T, (2020). CD81 Controls Beige Fat Progenitor Cell Growth and Energy Balance via FAK Signaling. Cell 182, 563–577.e20. 32615086 10.1016/j.cell.2020.06.021 PMC7415677

[93] Chao LC, Bensinger SJ, Villanueva CJ, Wroblewski K, and Tontonoz P (2008). Inhibition of adipocyte differentiation by Nur77, Nurr1, and Nor1. Mol. Endocrinol 22, 2596–2608. 18945812 10.1210/me.2008-0161 PMC2610364

[94] Cohen P, Levy JD, Zhang Y, Frontini A, Kolodin DP, Svensson KJ, Lo JC, Zeng X, Ye L, Khandekar MJ, (2014). Ablation of PRDM16 and beige adipose causes metabolic dysfunction and a subcutaneous to visceral fat switch. Cell 156, 304–316. 24439384 10.1016/j.cell.2013.12.021 PMC3922400

[95] Seale P, Conroe HM, Estall J, Kajimura S, Frontini A, Ishibashi J, Cohen P, Cinti S, and Spiegelman BM (2011). Prdm16 determines the thermogenic program of subcutaneous white adipose tissue in mice. J. Clin. Investig 121, 96–105. 21123942 10.1172/JCI44271 PMC3007155

[96] Adachi Y, Ueda K, Nomura S, Ito K, Katoh M, Katagiri M, Yamada S, Hashimoto M, Zhai B, Numata G, (2022). Beiging of perivascular adipose tissue regulates its inflammation and vascular remodeling. Nat. Commun 13, 5117. 36071032 10.1038/s41467-022-32658-6 PMC9452496

[97] Sidossis LS, Porter C, Saraf MK, Børsheim E, Radhakrishnan RS, Chao T, Ali A, Chondronikola M, Mlcak R, Finnerty CC, (2015). Browning of Subcutaneous White Adipose Tissue in Humans after Severe Adrenergic Stress. Cell Metab 22, 219–227. 26244931 10.1016/j.cmet.2015.06.022 PMC4541608

[98] Petruzzelli M, Schweiger M, Schreiber R, Campos-Olivas R, Tsoli M, Allen J, Swarbrick M, Rose-John S, Rincon M, Robertson G, (2014). A switch from white to brown fat increases energy expenditure in cancer-associated cachexia. Cell Metab 20, 433–447. 25043816 10.1016/j.cmet.2014.06.011

[99] Abdullahi A, and Jeschke MG (2017). Taming the Flames: Targeting White Adipose Tissue Browning in Hypermetabolic Conditions. Endocr. Rev 38, 538–549. 28938469 10.1210/er.2017-00163 PMC5716828

[100] Finlin BS, Memetimin H, Confides AL, Kasza I, Zhu B, Vekaria HJ, Harfmann B, Jones KA, Johnson ZR, Westgate PM, (2018). Human adipose beiging in response to cold and mirabegron. JCI Insight 3, e121510. 30089732 10.1172/jci.insight.121510 PMC6129119

[101] Seeliger C, Krauss T, Honecker J, Mengel LA, Buekens L, Mesas-Fernández A, Skurk T, Claussnitzer M, and Hauner H (2022). miR-375 is cold exposure sensitive and drives thermogenesis in visceral adipose tissue derived stem cells. Sci. Rep 12, 9557. 35688898 10.1038/s41598-022-13610-6 PMC9187663

[102] Backdahl J, Franzén L, Massier L, Li Q, Jalkanen J, Gao H, Andersson A, Bhalla N, Thorell A, Rydén M, (2021). Spatial mapping reveals human adipocyte subpopulations with distinct sensitivities to insulin. Cell Metab 33, 1869–1882.e6. 34380013 10.1016/j.cmet.2021.07.018

[103] Lazarescu O, Ziv-Agam M, Haim Y, Hekselman I, Jubran J, Shneyour A, Muallem H, Zemer A, Rosengarten-Levin M, Kitsberg D, (2025). Human subcutaneous and visceral adipocyte atlases uncover classical and nonclassical adipocytes and depot-specific patterns. Nat. Genet 57, 413–426. 39856219 10.1038/s41588-024-02048-3 PMC11821520

[104] Sun W, Dong H, Balaz M, Slyper M, Drokhlyansky E, Colleluori G, Giordano A, Kovanicova Z, Stefanicka P, Balazova L, (2020). snRNA-seq reveals a subpopulation of adipocytes that regulates thermogenesis. Nature 587, 98–102. 33116305 10.1038/s41586-020-2856-x

[105] Schaum N, Lehallier B, Hahn O, Pálovics R, Hosseinzadeh S, Lee SE, Sit R, Lee DP, Losada PM, Zardeneta ME, (2020). Ageing hallmarks exhibit organ-specific temporal signatures. Nature 583, 596–602. 32669715 10.1038/s41586-020-2499-y PMC7757734

[106] Zhou Q, Wan Q, Jiang Y, Liu J, Qiang L, and Sun L (2020). A Landscape of Murine Long Non-Coding RNAs Reveals the Leading Transcriptome Alterations in Adipose Tissue during Aging. Cell Rep 31, 107694. 32460027 10.1016/j.celrep.2020.107694 PMC7603645

[107] Whytock KL, Divoux A, Sun Y, Pino MF, Yu G, Jin CA, Robino JJ, Plekhanov A, Varlamov O, Smith SR, (2024). Aging human abdominal subcutaneous white adipose tissue at single cell resolution. Aging Cell 23, e14287. 39141531 10.1111/acel.14287 PMC11561672

[108] Chumlea WC, Rhyne RL, Garry PJ, and Hunt WC (1989). Changes in anthropometric indices of body composition with age in a healthy elderly population. Am. J. Hum. Biol 1, 457–462. 28514105 10.1002/ajhb.1310010408

[109] Yu L, Wan Q, Liu Q, Fan Y, Zhou Q, Skowronski AA, Wang S, Shao Z, Liao CY, Ding L, (2024). IgG is an aging factor that drives adipose tissue fibrosis and metabolic decline. Cell Metab 36, 793–807.e5. 38378001 10.1016/j.cmet.2024.01.015 PMC11070064

[110] Alba DL, Farooq JA, Lin MYC, Schafer AL, Shepherd J, and Koliwad SK (2018). Subcutaneous Fat Fibrosis Links Obesity to Insulin Resistance in Chinese Americans. J. Clin. Endocrinol. Metab 103, 3194–3204. 29846621 10.1210/jc.2017-02301 PMC6126891

[111] Caso G, McNurlan MA, Mileva I, Zemlyak A, Mynarcik DC, and Gelato MC (2013). Peripheral fat loss and decline in adipogenesis in older humans. Metabolism 62, 337–340. 22999012 10.1016/j.metabol.2012.08.007 PMC3531563

[112] Berry DC, Jiang Y, Arpke RW, Close EL, Uchida A, Reading D, Berglund ED, Kyba M, and Graff JM (2017). Cellular Aging Contributes to Failure of Cold-Induced Beige Adipocyte Formation in Old Mice and Humans. Cell Metab 25, 481. 28178569 10.1016/j.cmet.2017.01.011

[113] Han H-S, Ahn E, Park ES, Huh T, Choi S, Kwon Y, Choi BH, Lee J, Choi YH, Jeong YL, (2023). Impaired BCAA catabolism in adipose tissues promotes age-associated metabolic derangement. Nat. Aging 3, 982–1000. 37488415 10.1038/s43587-023-00460-8

[114] Alt EU, Senst C, Murthy SN, Slakey DP, Dupin CL, Chaffin AE, Kadowitz PJ, and Izadpanah R (2012). Aging alters tissue resident mesenchymal stem cell properties. Stem Cell Res 8, 215–225. 22265741 10.1016/j.scr.2011.11.002

[115] Karagiannides I, Tchkonia T, Dobson DE, Steppan CM, Cummins P, Chan G, Salvatori K, Hadzopoulou-Cladaras M, and Kirkland JL (2001). Altered expression of C/EBP family members results in decreased adipogenesis with aging. Am. J. Physiol. Regul. Integr. Comp. Physiol 280, R1772–R1780. 11353682 10.1152/ajpregu.2001.280.6.R1772

[116] Tchkonia T, Morbeck DE, Von Zglinicki T, Van Deursen J, Lustgarten J, Scrable H, Khosla S, Jensen MD, and Kirkland JL (2010). Fat tissue, aging, and cellular senescence. Aging Cell 9, 667–684. 20701600 10.1111/j.1474-9726.2010.00608.x PMC2941545

[117] Huang W, Hickson LJ, Eirin A, Kirkland JL, and Lerman LO (2022). Cellular senescence: the good, the bad and the unknown. Nat. Rev. Nephrol 18, 611–627. 35922662 10.1038/s41581-022-00601-z PMC9362342

[118] Xu M, Tchkonia T, Ding H, Ogrodnik M, Lubbers ER, Pirtskhalava T, White TA, Johnson KO, Stout MB, Mezera V, (2015). JAK inhibition alleviates the cellular senescence-associated secretory phenotype and frailty in old age. Proc. Natl. Acad. Sci. USA 112, E6301–E6310. 26578790 10.1073/pnas.1515386112 PMC4655580

[119] Suda M, Shimizu I, Katsuumi G, Yoshida Y, Hayashi Y, Ikegami R, Matsumoto N, Yoshida Y, Mikawa R, Katayama A, (2021). Senolytic vaccination improves normal and pathological age-related phenotypes and increases lifespan in progeroid mice. Nat. Aging 1, 1117–1126. 37117524 10.1038/s43587-021-00151-2

[120] Xu M, Palmer AK, Ding H, Weivoda MM, Pirtskhalava T, White TA, Sepe A, Johnson KO, Stout MB, Giorgadze N, (2015). Targeting senescent cells enhances adipogenesis and metabolic function in old age. eLife 4, e12997. 26687007 10.7554/eLife.12997 PMC4758946

[121] Zhang Z, Schaefer C, Jiang W, Lu Z, Lee J, Sziraki A, Abdulraouf A, Wick B, Haeussler M, Li Z, (2025). A panoramic view of cell population dynamics in mammalian aging. Science 387, eadn3949. 39607904 10.1126/science.adn3949 PMC11910726

[122] Wu D, Ren Z, Pae M, Guo W, Cui X, Merrill AH, and Meydani SN (2007). Aging up-regulates expression of inflammatory mediators in mouse adipose tissue. J. Immunol 179, 4829–4839. 17878382 10.4049/jimmunol.179.7.4829

[123] Tchkonia T, Pirtskhalava T, Thomou T, Cartwright MJ, Wise B, Karagiannides I, Shpilman A, Lash TL, Becherer JD, and Kirkland JL (2007). Increased TNFalpha and CCAAT/enhancer-binding protein homologous protein with aging predispose preadipocytes to resist adipogenesis. Am. J. Physiol. Endocrinol. Metab 293, E1810–E1819. 17911345 10.1152/ajpendo.00295.2007

[124] Hotamisligil GS, Peraldi P, Budavari A, Ellis R, White MF, and Spiegelman BM (1996). IRS-1-mediated inhibition of insulin receptor tyrosine kinase activity in TNF-alpha- and obesity-induced insulin resistance. Science 271, 665–668. 8571133 10.1126/science.271.5249.665

[125] Rogers NH, Landa A, Park S, and Smith RG (2012). Aging leads to a programmed loss of brown adipocytes in murine subcutaneous white adipose tissue. Aging Cell 11, 1074–1083. 23020201 10.1111/acel.12010 PMC3839316

[126] Gustafson B, Nerstedt A, and Smith U (2019). Reduced subcutaneous adipogenesis in human hypertrophic obesity is linked to senescent precursor cells. Nat. Commun 10, 2757. 31227697 10.1038/s41467-019-10688-x PMC6588633

[127] Espinosa De Ycaza AE, Søndergaard E, Morgan-Bathke M, Carranza Leon BG, Lytle KA, Ramos P, Kirkland JL, Tchkonia T, and Jensen MD (2021). Senescent cells in human adipose tissue: A cross-sectional study. Obesity 29, 1320–1327. 34114359 10.1002/oby.23202 PMC8859802

[128] Baker DJ, Perez-Terzic C, Jin F, Pitel KS, Niederländer NJ, Jeganathan K, Yamada S, Reyes S, Rowe L, Hiddinga HJ, (2008). Opposing roles for p16Ink4a and p19Arf in senescence and ageing caused by BubR1 insufficiency. Nat. Cell Biol 10, 825–836. 18516091 10.1038/ncb1744 PMC2594014

[129] Baker DJ, Wijshake T, Tchkonia T, LeBrasseur NK, Childs BG, van de Sluis B, Kirkland JL, and van Deursen JM (2011). Clearance of p16Ink4a-positive senescent cells delays ageing-associated disorders. Nature 479, 232–236. 22048312 10.1038/nature10600 PMC3468323

[130] Grosse L, Wagner N, Emelyanov A, Molina C, Lacas-Gervais S, Wagner KD, and Bulavin DV (2020). Defined p16(High) Senescent Cell Types Are Indispensable for Mouse Healthspan. Cell Metab 32, 87–99.e6. 32485135 10.1016/j.cmet.2020.05.002

[131] Colon-Mesa I, Fernández-Galilea M, Sáinz N, Lopez-Yus M, Artigas JM, Arbonés-Mainar JM, Félix-Soriano E, Escoté X, and Moreno-Aliaga MJ (2021). Regulation of p27 and Cdk2 Expression in Different Adipose Tissue Depots in Aging and Obesity. Int. J. Mol. Sci 22, 11745. 34769201 10.3390/ijms222111745 PMC8584112

[132] Morrison RF, and Farmer SR (1999). Role of PPARgamma in regulating a cascade expression of cyclin-dependent kinase inhibitors, p18(INK4c) and p21(Waf1/Cip1), during adipogenesis. J. Biol. Chem 274, 17088–17097. 10358062 10.1074/jbc.274.24.17088

[133] Sawada D, Kato H, Kaneko H, Kinoshita D, Funayama S, Minamizuka T, Takasaki A, Igarashi K, Koshizaka M, Takada-Watanabe A, (2023). Senescence-associated inflammation and inhibition of adipogenesis in subcutaneous fat in Werner syndrome. Aging (Albany NY) 15, 9948–9964. 37793000 10.18632/aging.205078 PMC10599740

[134] Le Pelletier L, Mantecon M, Gorwood J, Auclair M, Foresti R, Motterlini R, Laforge M, Atlan M, Fève B, Capeau J, (2021). Metformin alleviates stress-induced cellular senescence of aging human adipose stromal cells and the ensuing adipocyte dysfunction. eLife 10, e62635. 34544550 10.7554/eLife.62635 PMC8526089

[135] Zaragosi LE, Wdziekonski B, Villageois P, Keophiphath M, Maumus M, Tchkonia T, Bourlier V, Mohsen-Kanson T, Ladoux A, Elabd C, (2010). Activin a plays a critical role in proliferation and differentiation of human adipose progenitors. Diabetes 59, 2513–2521. 20530742 10.2337/db10-0013 PMC3279533

[136] Kuilman T, Michaloglou C, Vredeveld LCW, Douma S, van Doorn R, Desmet CJ, Aarden LA, Mooi WJ, and Peeper DS (2008). Oncogene-induced senescence relayed by an interleukin-dependent inflammatory network. Cell 133, 1019–1031. 18555778 10.1016/j.cell.2008.03.039

[137] Gao P, Jiang Y, Wu H, Sun F, Li Y, He H, Wang B, Lu Z, Hu Y, Wei X, (2020). Inhibition of Mitochondrial Calcium Overload by SIRT3 Prevents Obesity- or Age-Related Whitening of Brown Adipose Tissue. Diabetes 69, 165–180. 31712319 10.2337/db19-0526

[138] Shuai L, Zhang LN, Li BH, Tang CL, Wu LY, Li J, and Li JY (2019). SIRT5 Regulates Brown Adipocyte Differentiation and Browning of Subcutaneous White Adipose Tissue. Diabetes 68, 1449–1461. 31010955 10.2337/db18-1103

[139] Wronska A, Lawniczak A, Wierzbicki PM, and Kmiec Z (2016). Age-Related Changes in Sirtuin 7 Expression in Calorie-Restricted and Refed Rats. Gerontology 62, 304–310. 26595207 10.1159/000441603

[140] Fang J, Ianni A, Smolka C, Vakhrusheva O, Nolte H, Krüger M, Wietelmann A, Simonet NG, Adrian-Segarra JM, Vaquero A, (2017). Sirt7 promotes adipogenesis in the mouse by inhibiting autocatalytic activation of Sirt1. Proc. Natl. Acad. Sci. USA 114, E8352–E8361. 28923965 10.1073/pnas.1706945114 PMC5635888

[141] Picard F, Kurtev M, Chung N, Topark-Ngarm A, Senawong T, Machado De Oliveira R, Leid M, McBurney MW, and Guarente L (2004). Sirt1 promotes fat mobilization in white adipocytes by repressing PPAR-gamma. Nature 429, 771–776. 15175761 10.1038/nature02583 PMC2820247

[142] Majeed Y, Halabi N, Madani AY, Engelke R, Bhagwat AM, Abdesselem H, Agha MV, Vakayil M, Courjaret R, Goswami N, (2021). SIRT1 promotes lipid metabolism and mitochondrial biogenesis in adipocytes and coordinates adipogenesis by targeting key enzymatic pathways. Sci. Rep 11, 8177. 33854178 10.1038/s41598-021-87759-x PMC8046990

[143] Bernhardt A, Jamil A, Morshed MT, Ponnath P, Gille V, Stephan N, Sauer H, and Wartenberg M (2024). Oxidative stress and regulation of adipogenic differentiation capacity by sirtuins in adipose stem cells derived from female patients of advancing age. Sci. Rep 14, 19885. 39191852 10.1038/s41598-024-70382-x PMC11349916

[144] Saito M, Okamatsu-Ogura Y, Matsushita M, Watanabe K, Yoneshiro T, Nio-Kobayashi J, Iwanaga T, Miyagawa M, Kameya T, Nakada K, (2009). High incidence of metabolically active brown adipose tissue in healthy adult humans: effects of cold exposure and adiposity. Diabetes 58, 1526–1531. 19401428 10.2337/db09-0530 PMC2699872

[145] Holman CD, Sakers AP, Calhoun RP, Cheng L, Fein EC, Jacobs C, Tsai L, Rosen ED, and Seale P (2024). Aging impairs cold-induced beige adipogenesis and adipocyte metabolic reprogramming. eLife 12. 10.7554/eLife.87756 PMC11111218 38775132

[146] Benvie AM, Lee D, Steiner BM, Xue S, Jiang Y, and Berry DC (2023). Age-dependent Pdgfrβ signaling drives adipocyte progenitor dysfunction to alter the beige adipogenic niche in male mice. Nat. Commun 14, 1806. 37002214 10.1038/s41467-023-37386-z PMC10066302

[147] Lee MW, Odegaard JI, Mukundan L, Qiu Y, Molofsky AB, Nussbaum JC, Yun K, Locksley RM, and Chawla A (2015). Activated type 2 innate lymphoid cells regulate beige fat biogenesis. Cell 160, 74–87. 25543153 10.1016/j.cell.2014.12.011 PMC4297518

[148] Goldberg EL, Shchukina I, Youm YH, Ryu S, Tsusaka T, Young KC, Camell CD, Dlugos T, Artyomov MN, and Dixit VD (2021). IL-33 causes thermogenic failure in aging by expanding dysfunctional adipose ILC2. Cell Metab 33, 2277–2287.e5. 34473956 10.1016/j.cmet.2021.08.004 PMC9067336

[149] Wang W, Ishibashi J, Trefely S, Shao M, Cowan AJ, Sakers A, Lim HW, O’Connor S, Doan MT, Cohen P, (2019). A PRDM16-Driven Metabolic Signal from Adipocytes Regulates Precursor Cell Fate. Cell Metab 30, 174–189.e5. 31155495 10.1016/j.cmet.2019.05.005 PMC6836679

[150] Wang Q, Li H, Tajima K, Verkerke ARP, Taxin ZH, Hou Z, Cole JB, Li F, Wong J, Abe I, (2022). Post-translational control of beige fat biogenesis by PRDM16 stabilization. Nature 609, 151–158. 35978186 10.1038/s41586-022-05067-4 PMC9433319

[151] Park J, Hu R, Qian Y, Xiong S, El-Sabbagh AS, Ibrahim M, Wang J, Xu Z, Chen Z, Song Q, (2024). Estrogen counteracts age-related decline in beige adipogenesis through the NAMPT-regulated ER stress response. Nat. Aging 4, 839–853. 38858606 10.1038/s43587-024-00633-z PMC11829733

[152] Hasegawa Y, Ikeda K, Chen Y, Alba DL, Stifler D, Shinoda K, Hosono T, Maretich P, Yang Y, Ishigaki Y, (2018). Repression of Adipose Tissue Fibrosis through a PRDM16-GTF2IRD1 Complex Improves Systemic Glucose Homeostasis. Cell Metab 27, 180–194.e6. 29320702 10.1016/j.cmet.2017.12.005 PMC5765755

